# Gut microbial diversity and function analysis of the final-instar larvae of *Protohermes xanthodes* (Megaloptera: Corydalidae)

**DOI:** 10.1093/jisesa/iead065

**Published:** 2023-08-09

**Authors:** Zaiyun Shen, Chengquan Cao, Xiaofeng Xia

**Affiliations:** State Key Laboratory of Ecological Pest Control for Fujian and Taiwan Crops, Institute of Applied Ecology, Fujian Agriculture and Forestry University, Fuzhou 350002, China; Ministerial and Provincial Joint Innovation Centre for Safety Production of Cross-Strait Crops, Fujian Agriculture and Forestry University, Fuzhou 350002, China; Joint International Research Laboratory of Ecological Pest Control, Ministry of Education, Fuzhou 350002, China; School of Life Science, Leshan Normal University, Sichuan 614000, China; State Key Laboratory of Ecological Pest Control for Fujian and Taiwan Crops, Institute of Applied Ecology, Fujian Agriculture and Forestry University, Fuzhou 350002, China; Ministerial and Provincial Joint Innovation Centre for Safety Production of Cross-Strait Crops, Fujian Agriculture and Forestry University, Fuzhou 350002, China; Joint International Research Laboratory of Ecological Pest Control, Ministry of Education, Fuzhou 350002, China

**Keywords:** Megaloptera, *Protohermes xanthodes*, gut microorganism, function prediction

## Abstract

*Protohermes xanthodes* (Megaloptera, Corydalidae, *Protohermes*), widespread species in China, is an important insects treated as food and medicine for aquaculture. In this study, the gut microbiota was investigated by 16S rRNA microbial profiling. A total of 600 Amplicon Sequence Variants (ASV) were identified, Proteobacteria and Firmicutes were the main dominant phyla, and 27 genera ran through the entire digestive tract, mainly *Hafnia-Obesumbacterium* (Proteobacteria), *Lactobacillus* and *Lactococcus* (Firmicutes). The PICRUSt2 functional prediction of gut microbiota showed that the foregut abundant pathways related to metabolism, environmental information processing, and genetic information processing. while the midgut had the most abundant metabolic and environmental information processing pathways, including the prominent phosphotransferase system (PTS), propionate metabolism, and β-lactam resistance. The hindgut had the weakest metabolic function, but its genetic information processing was more abundant than the foregut and midgut. Additionally, 26 strains of bacteria were isolated from the midgut microorganisms, with Firmicutes being the dominant bacteria, and some of the purified bacteria had potential probiotic and anti-pathogen functions. These findings suggest that there are differences in the microorganisms of the different gut floras of the larvae, and each flora has specific metabolic functions. This research could be used to further understand the function of gut microorganisms, explore the co-evolution of *P. xanthodes* and gut microorganisms, and promote healthy breeding based on gut microorganisms.

## Introduction

The intestines of insects are colonized by a large number of microorganisms, and previous studies have revealed complex interactions between hosts and microbes. The composition of insect gut microbiota is affected by various factors such as host age, reproductive stage, diet, and environmental conditions (Kroetsch et al. 2020, Nobles and Jackson 2020). Research on gut microbes has demonstrated that gut microbiota plays a crucial role in host development, immunity, reproduction, and physiology (Minard et al. 2013, Coon et al. 2014, 2016, 2017). For example, the gut microbiota of *Drosophila melanogaster* promotes cell renewal and whole-body growth (Shin et al. 2011), microorganisms in the hindgut of termite can degrade cellulose and participate in the digestion of host food (Warnecke et al. 2007a), and the interactions between *Anopheles* and midgut bacteria are key to in the host immune response (Baton et al. 2009). The study of gut microbial diversity and function is helpful in understanding the physiological characteristics of insects, promoting the utilization of gut microorganisms for insect growth, and has directive significance for finding new methods of pest control and beneficial insect protection.

**Fig. 1.**
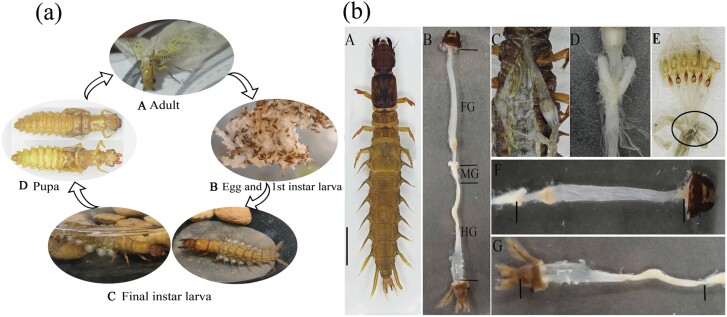
(a) Life history of *P. xanthodes*; (b) Structure of the alimentary canal the final-instar larva (a) A. adult; B. eggs and freshly hatched first-instar larvae; C. the final-instar larva in different postures in water; D. pupa dorsal and ventral surface; (b) A. the final-instar larva of *P. xanthodes*; B. digestive tract of the final-instar larva of *P. xanthodes*; C. anatomical diagram of the digestive tract; D. midgut; E. proventriculus and the cardiac valve; F. foregut; G. hindgut.

Characterization of microbial community structure is a prerequisite for understanding the role of microbial populations in the gut ecosystem. In recent years, with the development and application of new technologies such as molecular biology, metagenomics, and bioinformatics, the diversity, function, and application of insect gut microbiota have been extensively and deeply explored. Insights on the composition and key functions of insect gut microbiota are mainly from terrestrial insects (Douglas 2009, Engel and Moran 2013), while aquatic insects are neglected. Aquatic insects account for 60% of freshwater animal species (Ayayee et al. 2018), including 12 orders (Williams and Williams 2017, Hotaling et al. 2020, Zhao et al. 2021). *P. xanthodes* (Megaloptera: Corydalidae) is a holometabolous insect, with larvae living in water, pupae and adults being terrestrial (Fig. 1a). The larval stage accounts for most of the life cycle (Rasmussen and Pescador 2002), with 10–12 instars. Larvae are strict carnivores that prey on small aquatic animals such as the larvae of chironomids and caddis. *P. xanthodes* is a widely distributed species in China, and is a valuable species used as food and medicine insects for aquaculture. Its commercial and scientific value has attracted the attention of entrepreneurs and researchers. Most studies on the composition of the gut microbiome of freshwater insects focus on the disease-carrying Diptera, studying the interaction of its gut microbiome with pathogens (Moll et al. 2001, Wang et al. 2011, Chavshin et al. 2012). To the best of our knowledge, there have been few studies on the gut microbiome of the Megaloptera, only the gut bacteria of *Neochauliodes sparsus* larvae were isolated by traditional isolation and culture technology (Wang and Liu 2010), yielding 3 strains, all belonging to the *Bacillus*, which were *Bacillus megaterium*, *Bacillus subtilis*, and *Bacillus licheniformis*. The gut microorganism of the other Megaloptera has not been reported.

In recent years, the population of wild Megaloptera has seen a drastic decline. *P. xanthodes* is a dominant species in aquaculture, however, diseases are a major factor that is hindering its development, especially infect the final-instar larvae. Most of the pathogenic diseases in aquaculture are treated with antibiotics, while it has been observed that the *P. xanthodes* are almost no longer ingesting anything after infection, making it difficult to treat with antibiotics, and the use of antibiotics in aquaculture has faced limited success and potential danger. Therefore, it is of great significance to develop alternatives to antibiotics in aquaculture in order to effectively solve the problem of antibiotic resistance. Probiotics can be used to enhance the immune status and reduce the risk of disease.

The digestive tract of insects is divided into 3 parts: the foregut, midgut, and hindgut (Fig. 1b), including gula, food channel, crop, proventriculus, foregut, midgut, hindgut (ileum, colon, rectum). Each part has distinct structures and functions, such as digestion, absorption, and excretion. There are microbial communities driven by different metabolic processes in the digestive tract of insects (Nielsen et al. 2017). The foregut stores and predigested food, and microbacterium arborescens has been found in the foregut of *Spodoptera litura*, which produces N-acyl amino acid hydrolase and potentially affects the availability of nutrients by releasing amino acids (Ping et al. 2007). The hindgut is essential for the reabsorption of nutrients and ions. Studies have shown that the gut microbiota in the termite hindgut has a degradation effect on cellulose (Warnecke et al. 2007b); and is also found to have rich cell movement, energy metabolism, metabolic functions of cofactors and vitamins (Chew et al. 2018). The midgut is the primary site of insect digestion and absorption, inhabiting a large number of microorganisms, which are essential to the digestion, metabolism, development, and immunity of the host. For example, the termite midgut microbes can effectively degrade cellulose and help the host to digest food (Dar et al. 2022); while the midgut of vector mosquitoes serves as the first immune barrier against the invasion of external pathogens, with some gut microorganisms regulating the spread of pathogens in mosquitoes by secreting substances (Whitten et al. 2006). As gut microbiomes are complex and dynamic communities with a profound influence on the host, it is important to systematically characterize the bacterial communities in different gut floras. The relationship between the gut microorganisms of *P. xanthodes* and its hosts needs to be further explored.

In the present study, 16S rRNA microbial profiling was used to analyze the different floras of the digestive tract of the final-instar larvae of *P. xanthodes* for the first time, and culturable microorganisms were isolated and purified from the midgut of the final-instar larvae using traditional isolation and culture methods. The main objective of this study was to investigate the microbial structure and functional characteristics of different gut floras, and to evaluate their potential role in physiological processes such as nutrient metabolism, growth and development, immune regulation of *P. xanthodes*, laying the foundation for the subsequent utilization of gut microorganisms of *P. xanthodes* and promoting its healthy aquaculture.

## Materials and Methods

### Sample Acquisition and Processing

The final-instar larvae of *P. xanthodes* were collected from Emei River, Fuxi Town, Emeishan City, Leshan City, Sichuan Province (103°33ʹ58.04ʺE, 29°36ʹ20.05ʺ N, Altitude 390 m) in March 2022. The samples were brought back to the laboratory for normal breeding to adapt to the conditions in the laboratory. During the breeding period, the samples were normally fed for 2 wk, then the feeding began to decline. When sampling, the samples with consistent growth stages which consume very little food were selected for subsequent study.

### Sample Collection

Fifteen final-instar larvae of *P. xanthodes* with good growth conditions after starvation for 24 h were randomly divided into 3 groups. The larvae were washed 3 times with sterile water and disinfected with 75% alcohol for 3 min on an ultra-clean bench sterilized by ultraviolet for 30 min. After rinsing with distilled water, the samples were dried with sterile paper and the entire digestive tract was taken out in a sterile petri dish, the intestines of the samples were dissected under sterile conditions, the entire digestive tract was cut from the end of the abdomen along the midline of the back with sterile scissors, and the foregut, midgut, and hindgut were intercepted and placed in a sterile 1.5 ml centrifuge tube after surface tissues of each intestinal segment were washed with sterile water. Five samples were labeled on the tube and stored at −80°C.

### DNA Extraction and 16S rRNA Sequencing

Total DNA was extracted with E.Z.N.A. SOIL2.2 (Omega Bio-Tek. Norcross, GA. USA) and subjected to 16S rRNA sequencing. The hypervariable region V3-V4 of 16S rRNA gene was amplified with upstream primer 338F (5’-ACTCCTACGGGAGGCAGCAG-3’) and downstream primer 806R (5’-GGACTACHVGGGTWTCTAAT-3’) (Mori et al. 2014, Liu et al. 2016). The Polymerase chain reactions (PCR) reaction was performed in a volume of 20 μl, including 4 μl of 5× FastPfu Buffer, 2 μl of 2.5 mM dNTPs, 0.8 μl of Forward Primer (5 μM), 0.8 μl of Reverse Primer (5 μM), 0.4 μl of FastPfu Polymerase, 0.2 μl of BSA, 10 ng of DNA template, and ddH_2_O to 20 μl. 3 min at 95°C, 30 s at 95°C; 30 S at 55°C; single extension at 72°C for 10 min at 45 S. The PCR products were detected by 2% agarose gel electrophoresis.

### Bioinformatics Analysis

The hypervariable region V3-V4 of the 16S rRNA gene was sequenced using the Illumina Miseq PE300 platform (Illumina, San Diego, CA) at Majorbio Bio-Pharm Technology Co. Ltd. in Shanghai, China, through high-throughput sequencing. MiSeq sequencing obtained double-ended sequence data. Firstly, according to the overlapping relationship between PE reads, the paired reads were spliced into a sequence. At the same time, the quality of reads and the effect of merge were filtered by quality control. According to the barcode and primer sequences at the beginning and end of the sequence, the effective sequence was obtained and the sequence direction was corrected. FASTP (Chen et al. 2018) was used to control the quality of the double-ended original sequence, and Flash (Tanja et al. 2011) was used for splicing. The data removal methods and parameters are as follows: (i) The bases with a tail mass value of less than 20 were filtered, and a 50 bp window was set. If the average mass value in the window was less than 20, the back-end bases were cut off from the window, and the reads below 50 bp after quality control were filtered to remove the reads containing N bases. (ii) According to the overlapping relationship between PE reads, the paired reads were spliced into a sequence, and the minimum overlap length was 10 bp. (iii) The maximum mismatch ratio allowed by the overlap region of the spliced sequence is 0.2, and the nonconforming sequence is screened. (iv) According to the barcode and primers at the beginning and end of the sequence, the samples were distinguished and the sequence direction was adjusted. The barcode allowed the mismatch number to be 0, and the maximum primer mismatch number was 2.

The raw data were submitted to the National Center for Biotechnology Information (NCBI) sequence read archive (SRA) database. (SAMN31340536–SAMN31340544).

### Statistical Analysis

Sequence analysis was performed using the “Quantitative Insights into Microbial Ecology” pipeline (Qiime2 2020.2.0 version) (Bolyen et al. 2019), and additional analysis was performed using R (version 3.5.1) software. Multiplexed single-reads (a total of 460, 498) were imported into Qiime2. The “split amplicon denoising algorithm” “DADA2” (Callahan et al. 2016) plug-in in Qiime2 was used to “denois” sequencing readings. This step filters out the noise in the edge sequence and corrects the error, and removes the chimeric sequence and the singleton sequence. Finally, eliminates the obtained sequence, resulting in high-resolution expansion subsequence variants, amplicon sequence variants (ASVs), for downstream analysis. The common sequences of ASVs were classified using the classified-sklearn classifier trained according to the Silva 16S rRNA gene reference (Release 132) database (Quast et al. 2013). In order to complete the downstream diversity and composition analysis, the sequence was flattened to the minimum number of sequences per sample (*n* = 18, 110 sequences).

All data analysis was performed on the Meiji Biocloud platform (https://cloud.majorbio.com), as follows: Mothur 1.30 calculated the dilution curve and alpha diversity index, including observed richness (SOBs), Shannon index, and Chao1 index (Schloss et al. 2009). The Kruskal–Wallis H test was employed to assess the differences in alpha diversity between groups. Principal component analysis (PCA) based on Analysis of similarities (ANOSIM) (1,000 permutations) was performed to determine the dissimilarities in microbial community composition and to test their significance. Additionally, the Kruskal–Wallis H test was used to identify bacterial groups that showed significant differences in abundance at the phylum and genus levels among different groups. False Discovery Rate (FDR) correction was applied, and posthoc tests using Tukey–Kramer (0.95) were conducted to detect differences between multiple sample groups that showed differences. The gut microbiota potential functions of the samples were predicted by using PICRUST2 (Phylogenetic Investigation of Communities by Reconstruction of Unobserved States), a bioinformatics tool for comparing and predicting functional properties of microbial communities (Douglas et al. 2019, 2020, Wang et al. 2021, Hao et al. 2022). PICRUST2 was used to predict the potential functions of the gut microbiota of samples (Langille et al. 2013). In addition, One-way ANOVA was employed to compare the relative abundance of gut microbiota function at Kyoto encyclopedia of genes and genomes (KEGG) pathways with significant differences of gut flores.

### Isolation and Culture of Midgut Bacteria

Fifteen final-instar larvae of *P. xanthodes* with good conditions after starvation for 24 h were randomly selected and divided into 3 groups, larvae were washed 3 times with sterile water and disinfected with 75% alcohol body surface for 3 min on an ultra-clean bench sterilized by ultraviolet for 30 min. The larvae were then rinsed with distilled water and the intestines of the final-instar larvae of *P. xanthodes* were dissected under sterile conditions. The entire digestive tract was cut along the dorsal midline with sterile scissors, and the midgut tissue was taken out and placed into a 2 ml centrifuge tube containing 1 ml of sterile water. A 1–2 diameter 5 mm sterile metal ball was added to the tube, and the tube was placed on an oscillator at 20 HZ/min for 3 min to shock and break down the gut tissue, resulting in a gut homogenate.

The extracted gut contents were diluted in 5 gradients of 10^−1^, 10^−2^, 10^−3^, 10^−4^, and 10^−5^, and the diluents of the gut homogenate were then coated on 4 media: MRS medium (*Lactobacillus* culture medium), M8330; EC medium (*Enterococcus* culture medium), HB0133-3; Salmonella-Shigella Agar (selective media for *Salmonella*); and Nutrient Agar (general media for bacteria) and Luria Bertani (general media for bacteria): 10 g peptone, 5 g yeast extract, 10 g sodium chloride, 15 g agar, dissolved in 1 liter sterile water, adjusted to pH 7.0; then incubated at 37°C. The plate was observed every 12 h to obtain the original bacterial strain. The isolates were classified according to the differences in colony size, color, and morphology. The colonies of different forms were purified on LB plates for more than 5 generations to obtain monoclonal strains. The purified strains were stored in a 50% glycerol solution in a refrigerator at −80°C. The obtained monoclonal strains were grown in 500 μl liquid LB medium for 2–3 h at 37°C.

The bacterial culture was used as a template to amplify the 16S rRNA sequence. The 16S rRNA universal primers 27F (5’-AGAGTTTGATCCTGGCTCAG-3’) and 1492R (5’–GGTTACCT TGTTACGACTT-3’) were used to clone the bacterial 16S rRNA.The PCR reaction system was 25 μl: ddH_2_O 8.5 μl, Mix 12.5 μl, DNA 2 μl, 27F (10 mmol/liter) and 1492R (10 mmol/liter) 1 μl each. PCR: 95°C 5 min, (94°C 30 s, 56°C 30 s, 72°C 1 min) × 30 cycles, 72°C 10 min, 1.0% agarose gel electrophoresis to detect amplification products.

### Phylogenetic Analysis of Midgut Bacteria

The 16S rRNA sequence obtained by sequencing was blasted with the NCBI GenBank database, and the related sequences of each strain were selected. The sequence alignment was performed using clustalX1.83. The MEGA6.0 (Stecher et al. 2013) software was used. The Neighbor-Joining algorithm was used to construct the phylogenetic tree (Saitou 1987), and the bootstrap test was used to test the reliability of the phylogenetic tree.

## Results

### Gut Microbial Diversity and Composition of the Final-Instar Larvae of *P. xanthodes*

**Fig. 2.**
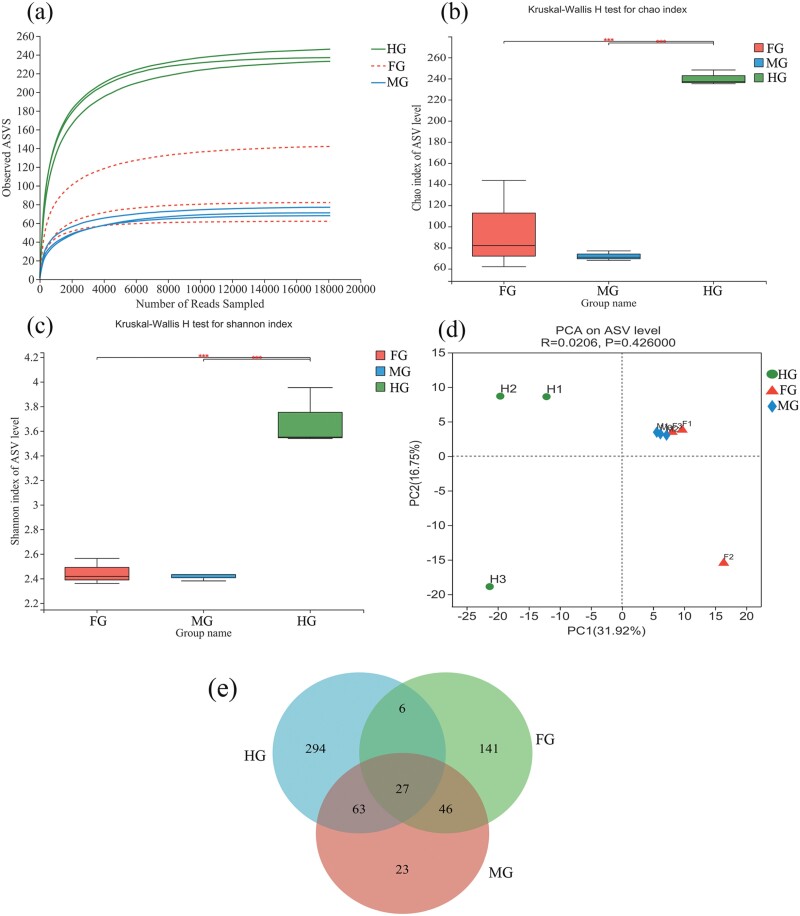
Diversity analysis of gut microbiota of the final-instar larva of *P. xanthodes.* Q, Z, and H are foregut, midgut, and hindgut of the final-instar larva of *P*. *xanthodes*. (a) Rarefaction curves based on Sobs values (the observed richness); (b) box diagram of Chao1 index; (c) box diagram of Shannon index; (d) PCA analysis of gut microbiota of the final-instar larva of *P*. *xanthodes*; (e) Venn diagram showing the distribution of ASV in intestine. The Kruskal–Wallis H test is employed to assess whether there is a significant difference between 3 groups. ***Statistically significant differences between the 2 groups being compared (*P* ≤ 0.001).

The rarefaction curve of all samples was flat, indicating that sequencing amount was sufficient to cover the species in all samples (Fig. 2a). Alpha diversity analysis revealed that the microbial diversity of the hindgut, foregut, and midgut of the final-instar larvae was significantly different (Kruskal–Wallis H, *P* < 0.001) (Fig. 2b and c), The Chao1 index and Shannon index of the foregut and midgut were similar (Kruskal–Wallis H, *P* > 0.05) (Fig. 2b and c), indicating that the species richness and community diversity of the foregut and midgut were comparable. Beta diversity assessment of the microbial composition in different regions of the intestine (Fig. 2d) showed that the microbial community in all samples could be divided into 3 distinct groups (ANOSIM, *R* = 0.0206, *P* = 0.426). The foregut (FG) and midgut (MG) were close, suggesting that the foregut and midgut had a similar microbial composition, and the hindgut microorganisms were significantly separated from the foregut and midgut.

A total of 19 phyla, 36 classes, 72 orders, 126 families, 177 genera, 219 species were detected in the gut samples of the final-instar larvae. The statistical results of species at different levels in the foregut, midgut, and hindgut showed in Supplementary Table S1. Of the 600 ASVs, 220 were in the foregut, 159 in the midgut, and 390 in the hindgut. To study the similarity of microbial composition in different sample tissues, Veen diagram analysis was conducted at the ASV level (Fig. 2e). There were 27 common core species present in all intestinal sections of the larvae. Additionally, 46 common species were found in the foregut and midgut, 6 species in the foregut and hindgut, 63 species in the midgut and hindgut, and 141, 23, 294 specific species were detected in foregut, midgut, and the hindgut, respectively.

**Fig. 3.**
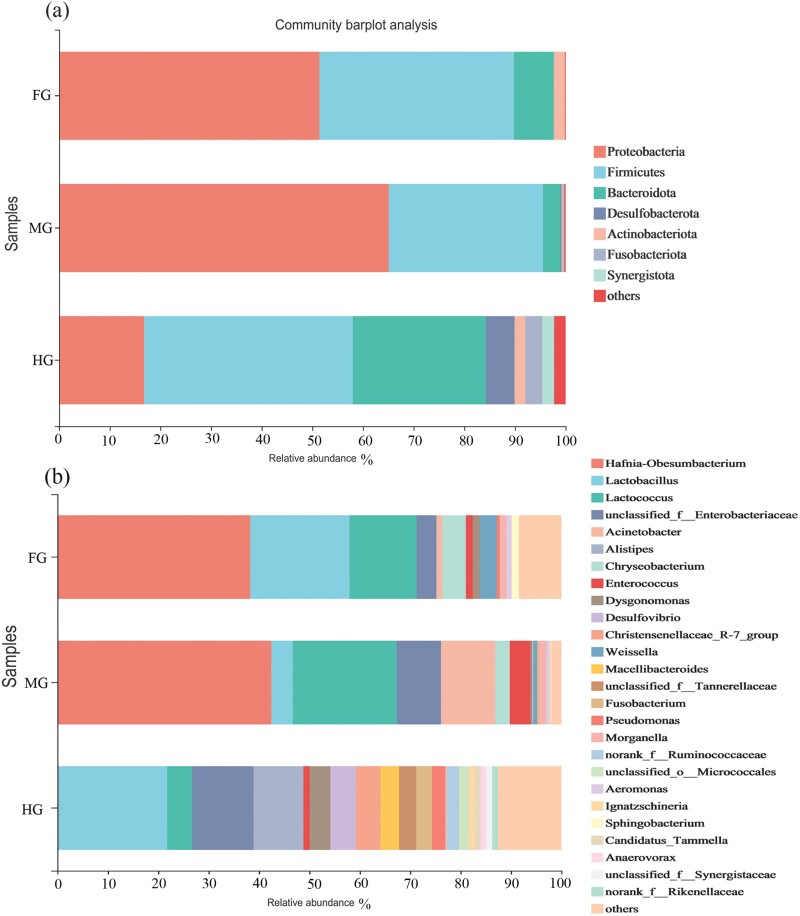
Gut microbial compositions at the phylum level and genus level of the final-instar larva. (a) Each bar represents average relative abundance of each phylum of gut microbiota in foregut (FG), midgut (MG), hindgut (HG). (b) Each bar represents average relative abundance of each genera of gut microbiota in foregut (FG), midgut (MG); hindgut (HG). The community bar plot shows the percentage abundance at different taxonomic levels, with species with relative abundance less than 1% represented by other species.

To further analyze the microbial composition characteristics of different intestines, the community composition of each sample was analyzed based on ASV at the phylum and genus levels (Fig. 3). Seven high-abundance phyla were detected in all gut samples, with the 3 most abundant phyla being Proteobacteria, Firmicutes, and Bacteroidota. At the phylum level, the dominant bacteria in the foregut were Proteobacteria (51.38%), Firmicutes (38.42%), Bacteroidota (7.86%), Actinobacteria (2.16%). The dominant phylum in the midgut was Proteobacteria (65.07%), Firmicutes (30.47%), and Bacteroidota (3.43%). The dominant phylum in hindgut was Firmicutes (41.19%), Bacteroidota (26.25%), Proteobacteria (16.78%), Desulfobacterota (5.69%), Fusobacteriota (3.36%), and Synergistota (2.32%). The unclassified phylum was more common in hindgut. The abundance of these microbial groups had undergone significant changes in the different gut floras. For example, Proteobacteria, Firmicutes were the dominant phylum in the foregut and midgut. The relative abundance of Proteobacteria in the foregut and midgut was higher than 50%, and Proteobacteria in the hindgut was reduced. The abundance of the dominant phylum Firmicutes, Bacteroidetes was higher than that in the foregut and midgut.

At the genus level (Fig. 3b), 26 high-abundance genera were detected in all samples. The genera with an average abundance of more than 1% were *Hafnia-Obesumbacterium*, *Lactobacillus*, *Lactococcus*, *Acinetobacter*, *Unclassified-Enterobacteriaceae*, and *Alistipes*. The top 5 dominant genera and abundances in each intestine were: foregut, *Hafnia-Obesumbacterium* (38.29%), *Lactobacillus* (19.68%), *Lactococcus* (13.32%), *Unclassfied-Enterobacteriaceae* (3.93%), *Chryseobacterium* (4.65%); in the midgut, *Hafnia-Obesumbacterium* (42.49%), *Lactococcus* (20.63%), *Acinetobacter* (10.75%), *unclassified_ f_Enterobacteriaceae* (8.77%), *Lactobacillus* (4.23%); In the hindgut, *Lactobacillus* (21.54%), *unclassified_f_Enterobacteriaceae* (12.18%), *Alistipes* (9.87%), *Desulfovibrio* (5.08%), *Lactococcus* (4.97%). The 2 dominant genera with the highest abundance in the foregut and midgut were *Hafnia-Obesumbacterium* and *Lactococcus*. The *Hafnia-Obesumbacterium* in the hindgut decreased sharply, and the *unclassified_ f_ Enterobacteriaceae* showed an increasing trend in the foregut, midgut, hindgut (Supplementary Fig. 1).

**Fig. 4.**
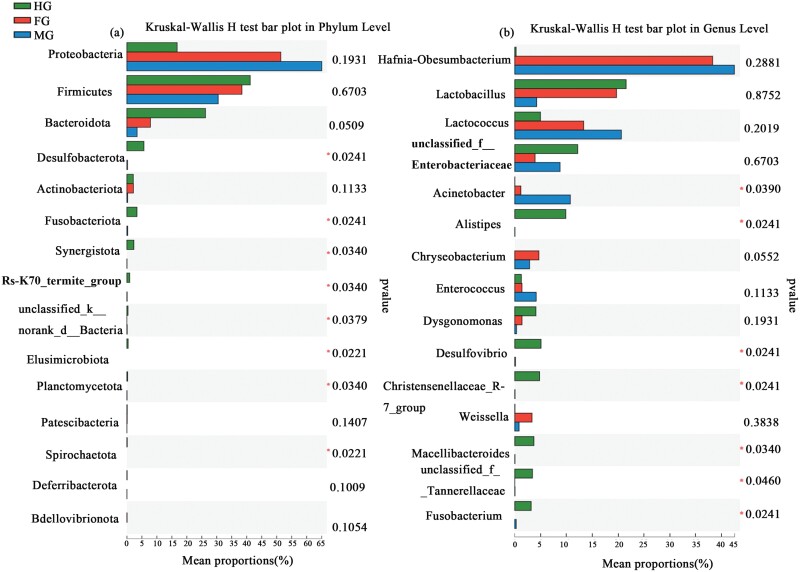
Top15 relative abundance distributions of (a) bacterial classes (phyla) and (b) genera (classes) assignable to ASVs recovered from the foregut (FG), midgut (MG), hindgut (HG) of the final-instar larva (Kruskal–Wallis H test). *Significant at 5% level of significance.

The differences between the samples were analyzed at the phylum and genus levels of the top 15 abundances (Fig. 4). There were significant differences (Kruskal–Wallis H test, *P* < 0.05) in Desulfovibrio, Fusobacteria, and Synergistota in the foregut, midgut, hindgut, and other low abundance phyla were also different (Fig. 4a) (Supplementary Table 2). The differences in Desulfobacterota, Fusobacteriota, Synergistota, Rs-K70_Termite_group, and Planctomycetota were mainly due to the fact that the abundance was high in the hindgut, none in the foregut, and low in the midgut. In addition, the Elusimicrobiota and Spirochaetota gates were only found in the hindgut; at the genus level, among the genera in higher abundance, *Acinetobacter*, *Alistipes*, and *Desulfovibrio* were significantly different (Kruskal–Wallis H test, *P* < 0.05) in the foregut, midgut, hindgut (Fig. 4b and Supplementary Table 3). Among them, *Alistipes*, *Christensenellaceae_R-7_group, Desulfovibrio*, *Macellibacteroides*, and *Fusobacterium* were different in 3 sections because they were high in the hindgut and none in the foregut, lower in midgut. *Acinetobacter* in the midgut was higher than that in the hindgut.

### Gut Microbial Function of the Final-Instar Larva of *P. xanthodes*

To compare the potential functions of microbial communities in different gut sections of the digestive tract at the ASV level, PICRUSt2 was utilized to predict the functions of gut microorganisms (Supplementary Fig. 2). At the KEGG 1-level pathway, the abundance was annotated to 6 KEGG1-level functional pathways, Metabolism 75.44%, Environmental information processing 7.32%, Genetic information processing 7.08%, Cell process 4.45%, Human disease 3.96%, and Organism system 1.75%, the abundant pathways were related to Metabolism, Environmental information processing and Genetic information processing. Specifically, in the foregut, rich pathways were related to metabolism, environmental information processing, and genetic information processing, the 3 pathways were equally abundant in the midgut, besides, the metabolic and environmental information processing pathways in the midgut were the most abundant in the 3 floras, hindgut metabolism accounts for the main part, genetic information processing was more abundant than environmental information processing, and was the only pathway beyond the foregut and midgut among all pathways in the hindgut.

Subsequently, 45 2-level KEGG pathways and 336 3-level KEGG pathways (Supplementary Fig. 2 and Supplementary Table S4) were predicted, and the rich differences in predictive functions between digestive tract floras were compared and analyzed. Based on one-way ANOVA (df = 2), it was found that KEGG pathways were significantly different (*P* < 0.05) in gut floras (Supplementary Fig. 2 and Supplementary Tables S5 and S6). The midgut was enriched in metabolisms, such as microbial metabolism, carbon metabolism, secondary metabolites, and amino acid synthesis in different environments. In addition, various pathways of carbohydrate metabolism, including the glycolytic pathway, pyruvate, and butyrate metabolism, were observed. The ABC transport and phosphotransferase system (PTS) and two-component system were abundant in environmental information processing. Among the differential pathways, the annotated carbohydrate, terpenoid, and polyketide metabolism, as well as amino acid metabolism (including propionate, phenylalanine, tyrosine, β-alanine, glutathione, glycolysis, and amino sugar nucleotide sugar metabolism) in the midgut were more prominent in the 3 floras. Furthermore, the bacterial community in the midgut had a significantly richer PTS, propionate metabolism, and β-lactam resistance.

The metabolic pathway also accounted for the main part of the foregut. The foregut and midgut annotated similar richness pathways (Supplementary Table S4). In terms of metabolism, the foregut showed a more abundant metabolism of thiamine, glycerol, and cyanamide than the midgut. The foregut also included biosynthesis of some substances such as streptomycin; the glucagon signaling pathway was enriched in biological systems. In the differential pathway, the foregut showed more abundant sugar biosynthesis and signal transduction functions.

The hindgut had the same metabolic pathways as the foregut and midgut, but the abundance decreases sharply (Supplementary Table S4). The hindgut microorganisms were highly enriched in genetic information processing such as ribose pathway and aminoacyl-tRNA biosynthesis. In addition, it also had rich sphingolipid metabolism, other glycan degradation, and sugar biosynthesis pathways.

### Isolation and Culture of Bacteria From the Midgut of the Final-Instar Larvae

**Fig. 5.**
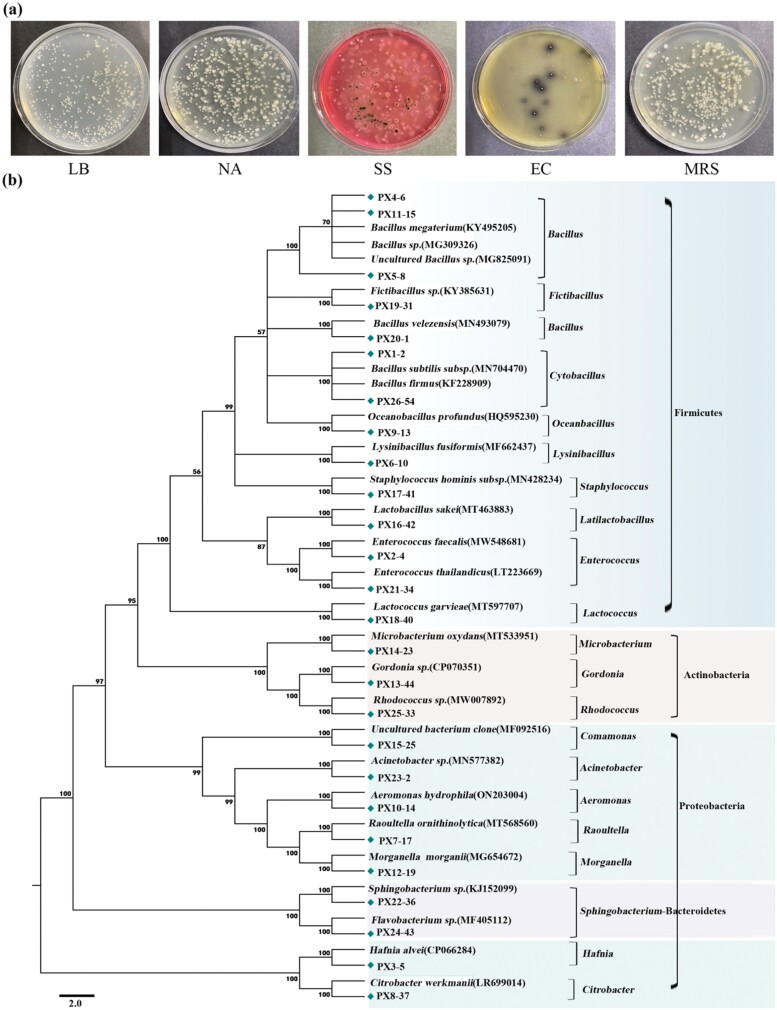
Phylogenetic analysis based on 16S rRNA sequences from the midgut of the final-instar larva of *P. xanthodes.* Sequences isolated from the midgut of the final-instar larva of *P. xanthodes* are marked with a diamond symbol. All positions containing gaps and missing data are eliminated. The bootstrap test (1,000 replicates) is shown next to the branches. The scale bar represents 2.0 estimated phylogenetic divergence.

The original strains of midgut were obtained on LB, NA, MRS, EC, and SS (Fig. 5), and 26 single bacterial colonies were isolated and purified (Supplementary Table 7). BLAST alignment combined with Ribosomal Database Project (RDP) Classifier analysis showed that 14 of the 26 strains of bacteria (PX4-6, PX11-15, PX5-8, PX19-31, PX20-1, PX1-2, PX26-52, PX9-13, PX6-10, PX17-41, PX16-42, PX2-4, PX21-34, PX18-40) isolated from the midgut belonged to Firmicutes. Other bacterial isolates identified as *Bacillus* (PX4-6, PX11-15, PX5-8, PX20-1), *Cytobacillus* (PX1-2, PX26-52), *Enterococcus* (PX2-4, PX21-34), *Fictibacillus* (PX19-31), *Oceanbacillus* (PX9-13), *Lysinibacillus* (PX6-10), *Staphylococcus* (PX17-41). *Latilactobacillus* (PX16-42), and *Lactococcus* (PX18-40).

7 strains of Proteobacteria (PX15-25, PX23-2, PX10-4, PX7-17, PX12-19, PX3-5, PX8-37) were isolated, containing the genus *Comamonas* (PX15-25); *Acinetobacter* (PX23-2); *Aeromonas* (PX10-4); *Raoultella* (PX7-17); *Hafnia* (PX3-5); *Morganella* (PX12-19); *Citrobacter* (PX8-37); 2 strains isolated from the genera *Sphingobacterium* (PX22-36, PX24-43); 3 strains belonged to Actinobacteria (PX14-23, PX13-44, PX25-33), containing the genus *Microbacterium* (PX14-23) *Gordonia* (PX13-44); *Rhodococcus* (PX25-33).

The phylogenetic analysis of the 16S rRNA of the midgut bacteria of the final-instar larva of *P. xanthodes* was carried out (Fig. 5). The phylogenetic tree further supported the RDP Classifier classification results. 26 strains were clustered into 4 family branches, of which Firmicutes was the most abundant family branch, indicating that Firmicutes was the dominant group in the midgut culturable bacteria of the final-instar larva of *P. xanthodes*, followed by Proteobacteria. This result is contrary to microbial profiling.

## Discussion

Gut microorganisms play an important role in maintaining the physiological homeostasis of the host. Currently, the exploration of gut microorganisms in aquatic insects is still limited. This paper studied the microbial diversity, composition, and potential function of the 3 flores of the final-instar larva of *P. xanthodes*. The results showed that the gut microbial diversity of the final-instar larva was abundant. Proteobacteria and Firmicutes were the 2 most important phyla, which were also the dominant phyla in the microorganisms of terrestrial invertebrates (Colman et al. 2012, Jones et al. 2013, Yun et al. 2014, Mikaelyan et al. 2015, Kim et al. 2017, Muturi et al. 2017). A study of 264 aquatic invertebrates found that Proteobacteria was the dominant phyla, accounting for 69.25% of the total sequence (Kroetsch et al. 2020). Similar dominant groups were also found in other freshwater invertebrates (Ayayee et al. 2018, Receveur et al. 2020). At the phylum level, the composition and abundance of the dominant bacteria in the foregut, midgut, and hindgut were different. For example, the abundance of Proteobacteria (foregut 51.38%, midgut 65.07%, hindgut 16.78%), Firmicutes (foregut 38.42%, midgut 30.47%, hindgut 41.19%), and Bacteroidetes (foregut 7.86%, midgut 3.43%, hindgut 26.52%) were varied in each gut segment. There were also significant differences in the abundance of Desulfobacterota, Fusobacteria, and Synergistota, and most of them were enriched in the hindgut. The high degree of enrichment of the 3 phyla bacteria may be related to their physiological functions in the host.

At the genus level, the dominant genera were *Hafnia-Obisumbacterium*, *Lactobacillus*, *Lactococcus*, *Acinetobacter*, *Unclassfied-Enterobacteriaceae*, *Alistipes*, etc. The genus *Hafnia- Obesumbacterium* was the most abundant dominant genus in the final-instar larva, with the highest abundance in the midgut (42.49%) and a sharp decrease in the hindgut (2.54%). There were few studies about this genus. It was found to be in high abundance in snakes that did not secrete venom (Qin et al. 2019), but its function was not studied. A study found that it could accelerate the loss of some dry matter and the hydrolysis of proteins (Wang et al. 2022), suggesting that such bacteria in the foregut and midgut were more likely to be involved in the digestion of some protein substances in the intestine. The second most abundant genus was *Lactobacillus*, which played a health-promoting role in the gastrogut tract through various mechanisms, such as pathogen elimination, maintenance of microbial balance, immune regulation, and other key functions (Yadav et al. 2017, Zhang et al. 2018). It also participated in the decomposition of sugars, the production of acids, and bacteriocins to protect the gut environment from decay and sulfate-reducing bacteria (Pessione 2012). Furthermore, *Lactobacillus* had a positive effect on the production of insect resistance (Xia et al. 2013), indicating that it may play an important role in host digestion, immunity, and protection of gut health. *Lactococcus* were found to be associated with fermentation and degradable cellulose (Ahn et al. 2012, Engel and Moran 2013). So the dominant genera may have important functions in the digestion, immunity, and other aspects of the *P. xanthodes*.

Among the digestive tract, 27 common genera as a core microbiota were presented in 3 flores, and 23, 8, 2 genera were shared by the midgut and hindgut, the foregut and midgut, the foregut and hindgut, respectively. More genera were shared between the midgut and hindgut, which may be due to the similarity of niche structure. Compared with the midgut and hindgut, the foregut lacked some genera, such as the *Alistipes* and *Desulfovibrio*, *Alistipes* is closely related to tryptophan metabolism, and the *Desulfovibrio* is known as sulfate-reducing bacteria. These bacteria may help the hindgut to recycle nutrients and inorganic salts. The *Lactococcus*, *Enterococcus*, *Acinetobacter* in the midgut were more abundant than in the foregut and hindgut, which helped the host to absorb nutrients and maintain health. The hindgut was mainly dominated by *Lactobacillus*. The difference in the microbial composition of 3 gut floras was related to the differentiation and specificity of their functions, suggesting a co-evolutionary relationship between the gut tract and the host.

There were 55 unique genera in the foregut. *Gordonia*, in high abundance, could degrade pyrene and produce carotenoids by fermentation, making it a potential organic pollutant-degrading bacteria that may degrade pollutants entering the digestive tract (Takaichi et al. 2008). The research on Microbacterium has revealed that it produces secondary metabolites with varying levels of antibacterial activity against bacteria and fungi (Lee et al. 2014, Kamjam et al. 2017), which could potentially help the host to ward off infection from certain pathogens. Particularly, *Leuconostoc* was only found in the midgut. *Leuconostoc* could maintain growth with carbohydrates as fermentation substrates, accompanied by the production of D-lactic acid, acetic acid, bacteriocin, extracellular polysaccharide, mannitol, diacetyl, and other metabolites. Some of these had obvious antagonistic effects on common pathogenic bacteria (Ruiz et al. 2018), and could also increase the activity of glutathione peroxidase and superoxide dismutase (Martinez et al. 2020), which was consistent with the related pathways predicted by midgut microbial function. The microbial composition of the foregut and midgut was similar. In addition to the genera mentioned above, there was a relatively rich *Acinetobacter*, which was also common in the intestines of other insects and had the function of assisting host food digestion and nitrogen transformation (Briones-Roblero et al. 2017). In addition, the hindgut had 53 independent genera, *g-unclassied-o-Micrococcales*, *g-unclassied-f-synergistaceae*, and *g-norank-f-Rickenellaceae* showed great abundance, although they are less studied.

In summary, our study suggests that the gut tract of *P. xanthodes* harbors potentially beneficial bacteria that may have positive effects on its growth and immunity. However, further research is needed to validate these findings and determine the exact mechanisms underlying these interactions. While our results provide valuable insights into the bacterial community of *P. xanthodes*, the functional roles of the identified bacteria remain speculative and require further investigation. Overall, our study represents an important initial exploration into the complex relationship between microbial communities and their host organisms. Future studies should focus on elucidating the specific mechanisms by which these bacteria influence *P. xanthodes*, and how these interactions can be leveraged for the development of novel therapeutic strategies.

The foregut and hindgut of the final-instar larvae are much longer than the midgut, and the midgut is thicker than the foregut and hindgut. The differences in morphological structure can lead to differences in microbial species by affecting the availability of nutrients, oxygen levels, and pH levels. The different structures of the gut can affect the types of bacteria that can survive in the gut, as well as the types of nutrients that are available for the bacteria to consume. The foregut is more acidic and has a higher pH than the midgut and hindgut, while the midgut is more alkaline than the foregut and hindgut. The hindgut is more anaerobic than the foregut and midgut. These differences in structure can affect the types of bacteria that can survive in the gut.

Alpha and beta diversity analyses indicated no significant difference between the foregut and midgut, but a significant difference was observed between the hindgut and both the foregut and midgut. The hindgut had the richest diversity of microbial species, followed by the foregut, and the midgut was the least, which was different from previous studies. Some studies about insect gut microbes found that the microbial diversity of the digestive tract was similar, and the microbial abundance of the foregut was higher than that of other gut segments (Douglas 2015, Chen et al. 2020), likely due to the fact that food has a significant impact on the gut microbial diversity, as microorganisms enter the gut with food, resulting in the microbial diversity of the foregut being higher than that of the midgut and hindgut as a short-term storage of food. However, there was a trend of decreasing microbial diversity and abundance in the foregut, midgut, and hindgut. The final-instar larvae are almost no longer eating, and there is almost no substance in the digestive tract, which is consistent with the results of gut anatomy. The microorganisms are mainly derived from the strains accumulated in the early stage of feeding and gut symbiosis, so the foregut microorganisms are not the highest. In addition, there are differences in the gut microbiota of insects at different life stages. The fourth-instar mosquito larvae after molting were found to have fewer microorganisms (Moll et al. 2001). *P. xanthodes* are holometabolous, the larvae rarely eat in the late stage of the final-instar, the pupae do not eat, and the adults only feed on little juice. Whether the gut microbiota of the final-instar larvae paves the way for the great changes in their habitat and feeding remains to be studied.

The potential functional capacities of microbial communities were distinctly different in gut flores and these differential microbial functional capacities are likely related to host physiological functions. PICRUST2 was used to predict the function of gut microflora. The most abundant functions of gut bacteria in larvae were metabolic pathways and biosynthesis of secondary metabolites. The protein/amino acid uptake system and catabolism in the gut microbiota are important metabolic processes that benefit both the gut microbiota and host insects. Many microbial species contain a variety of proteases involved in protein catabolism, such as *Fusobacterium*, *Bacteroides*, and *Lactobacillus* (Savijoki et al. 2006). The metabolic function abundance of foregut and midgut was similar. Additionally, the biosynthesis and metabolism of sugar and signal transduction showed greater abundance in the foregut. Mitogen-activated protein kinase (MAPK) is a group of evolutionarily conserved serine/threonine protein kinases that are activated by a series of extracellular stimuli and mediate signal transduction from the cell membrane to the nucleus (Yue and López 2020), moreover, the increased of insect hormone levels can activate the MAPK signaling pathway to trans-regulate the differential expression of multiple midgut receptor genes, resulting in high resistance of *Plutella xylostella* to *Bacillus thuringiensis* insecticidal protein Cry1Ac (Guo et al. 2020). It has revealed for the first time that insect hormones can participate in the new function of pest resistance, and clarified that hormone signal plasticity is a cross-border general strategy for organisms to resist the invasion of external pathogens.

The midgut is the main place for digestion and absorption, and it is also an important organ for maintaining body health. Midgut microorganisms showed powerful and rich functions. The bacterial community from the midgut had a more abundant PTS, propionate metabolism, β-lactam resistance, and other functions. Studies have shown that PTS not only controls nitrogen metabolism, and regulates the virulence of certain pathogens, but also mediates stress responses under pressure conditions (Deutscher et al. 2006); the metabolic function of the hindgut was weakened, but the cysteine, methionine, and PTS were strengthened. The biosynthesis and metabolism of sugar, lipid metabolism (sphingolipid metabolism) were enriched in the hindgut, meanwhile, the cell engineering (lysosome) and biological systems were also involved. Lipids are important substances to maintain ion balance, regulate biofilm structure and play an important role in reducing the osmotic shock of aquatic animals. The Glycerophospholipids are the main components of biofilms (Chen et al. 2014), and lipid metabolites produced by gut microorganisms are sources of carbon and energy storage for host insects. In addition, studies had found that there was a wide range of microbial fermentation of polysaccharides in the hindgut of insects, and a large amount of short-chain fatty acids were produced during fermentation (Zheng et al. 2017), which was related to the body weight of bees. The functions of these bacteria correspond to the functions of different gut floras, further suggesting the co-evolutionary relationship between gut microorganisms and hosts.

In the study of the interaction between the host and its gut microorganisms, the midgut microorganisms are key part, which is the main place for food digestion and metabolism. The PICRUST2 showed that the midgut microbe functions were rich and powerful, and their internal gut microbes had great potential for exploration. In this paper, 26 strains of bacteria were isolated from the midgut of the final-instar larvae by traditional isolation and culture methods. Among them, 14,7,2,3 strains belonged to Firmicutes, Proteobacteria, Bacteroidetes, and Actinobacteria. The dominant bacteria in the midgut were Firmicutes, which were mainly composed of *Bacillus*, *Cytobacillus*, *Enterococcus*, indicating that Firmicutes bacteria were easier to culture under aerobic conditions. Studies had shown that most of the bacteria isolated from the gut tract of aquatic invertebrates were facultative anaerobes (Klug and Kotarski 1980, Sugita et al. 1987, Santavy et al. 1990), with fewer aerobic and obligate anaerobes, which may be related to the aquatic environment. At the phylum level, the bacteria isolated and cultured in the midgut were opposite to the results of microbial profiling. The results of that showed that Proteobacteria was the most abundant, followed by Firmicutes. Microbial profiling can fully understand the bacteria in the environment and make up for the shortcomings of our traditional isolation and culture. The types of gut microorganisms are complex, most of them are uncultured, and it is also important to select the appropriate medium during isolation and culture. Among the isolated bacteria, *Bacillus* was the most abundant. Most species of *Bacillus* probiotics are capable of producing beneficial metabolites during growth, which can even enhance the host’s immunity to diseases (Hwang et al. 2012), making them widely applicable in practical production. At the same time, the midgut is also one of the important immune defense organs of insects. Gut microorganisms play a direct and indirect role in host immune defense. The dominant bacteria *Enterococcus* in the gut tract of *Spodoptera litura* larvae may be related to the colonization resistance of the host (Shao 2013). These studies suggest that the *Enterococcus* found in the gut tract of *P. xanthodes* may also play a role in its physiological process, and may be used as a potential gut probiotic to help *P. xanthodes* control diseases during breeding.

The 26 strains of bacteria that have been isolated have significant potential as microbial resources. The isolation of intestinal microorganisms is a crucial step in studying the relationship between microorganisms and their hosts, this can provide important resources for gaining a deeper understanding of intestinal microorganisms and their functions. We can investigate whether some bacterial strains can help insects digest food, enhance their immune system, or provide essential nutrients. These functions can have important effects on the insect’s lifespan and health, and studying whether the isolated bacteria play a role in the process of pathogen infection is also a promising research direction. In addition, some bacterial strains may affect the insect’s behavior, such as influencing its mating behavior or its ability to search for food. These are also important research directions because they can reveal the complex nature of insect-microbe symbiosis and provide insight into understanding ecosystems and biodiversity.

In this study, we found the diversity, composition, and function of gut microbiota in the final-instar larvae of *P.xanthodes*, as well as the differences in microbial species of different gut floras. At the same time, the microorganisms and their functions in different floras are highly consistent with the physiological functions of different gut floras, suggesting the co-evolutionary relationship between gut microbiota and host. This interaction between gut microbiota and the host is important for further study on the function of gut microbiota and the regulation of gut microbiota by the host. At the same time, this study also lays a good foundation for the development of gut microbiota of *P. xanthodes* to promote healthy aquaculture. Our study also has certain references and inspiration for the subsequent systematic study of the diversity and function of gut microorganisms in other aquatic insects.

It should be noted that we did not include a control (processed but with no gut tissue) to assess potential bacterial contaminants during sample preparation because the current experimental procedure is theoretically noninfectious. However, since there is a lack of effective negative controls, the possibility of contamination cannot be ruled out. In future experiments, we should design more reasonable negative controls to ensure the objectivity and accuracy of gut microbiome diversity. In addition, *P. xanthodes* lives in water and its food also lives in water. During the feeding process, it inevitably ingests bacteria in the water environment. However, in this study, the feeding of insects had basically stopped before the sample was dissected, and the insect was starved for 24 h before dissection, and our study also showed that the microbial composition of different segments of the intestine was different, thus, it can be considered that the bacteria in the gut are mainly resident bacteria. In the future, when we study aquatic insects, we should set more rigorous controls and more experiments to eliminate these possibilities.

## Supplementary Material

iead065_suppl_Supplementary_Material
