# Supplementary material for: Gut microbial diversity and function analysis of the final-instar larvae of Protohermes xanthodes (Megaloptera: Corydalidae)
Source: J Insect Sci. 2023 Aug 9;23(4):16. doi: 10.1093/jisesa/iead065 (PMC10411046; doi:10.1093/jisesa/iead065)
Supplement: iead065_suppl_Supplementary_Material [file iead065_suppl_supplementary_material.docx]

**Supplementary Materials**

Gut microbial diversity and function analysis of the final-instar larvae of *Protohermes xanthodes*

**This file includes:**

Supplementary Figures 1-2

Supplementary Tables 1-7


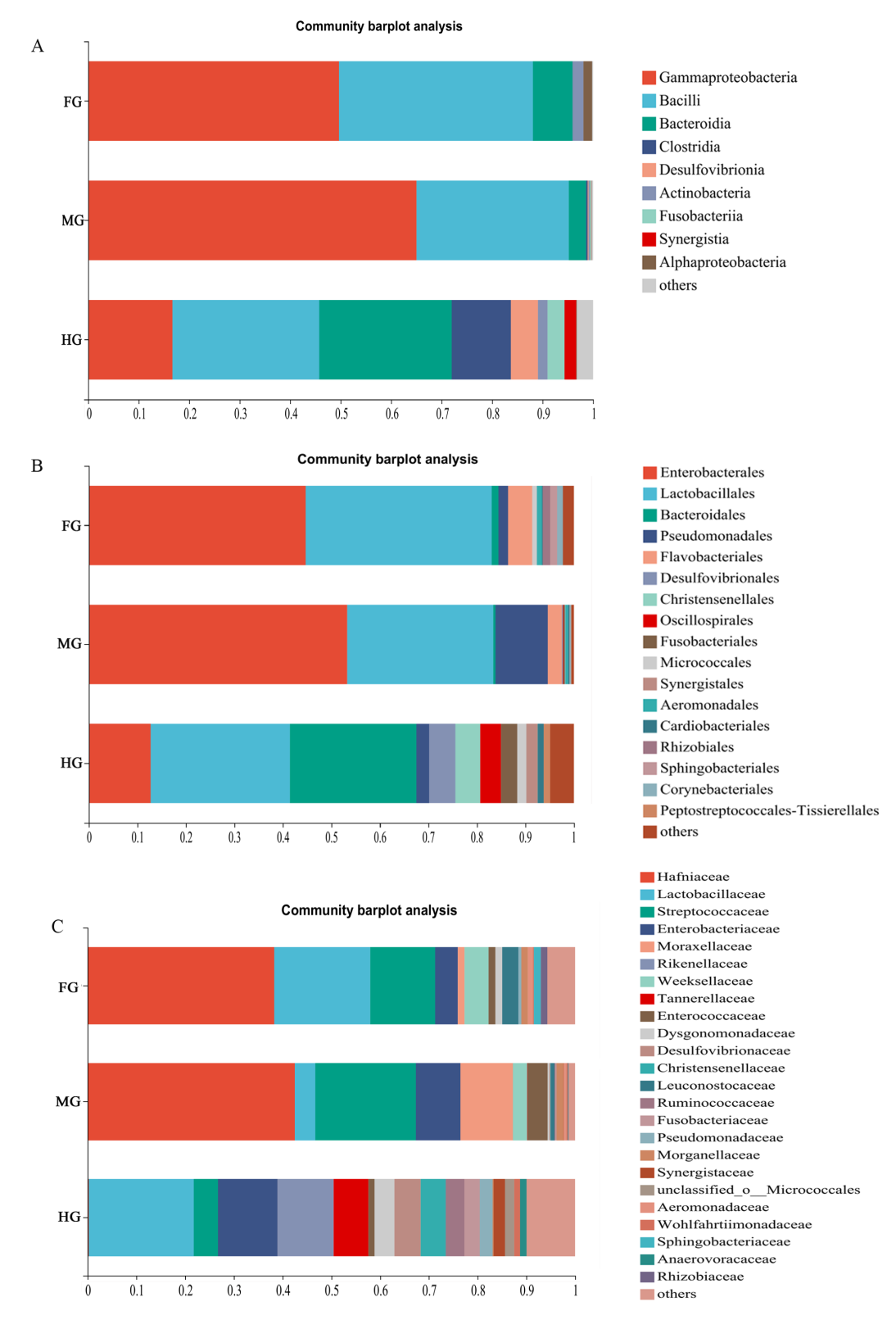


**Supplementary Figure 1** Gut microbial compositions of the final-instar larva.

A: Each bar represents average relative abundance of each Class of gut microbiota in foregut (FG), midgut (MG), hindgut (HG). B: Each bar represents average relative abundance of each Order of gut microbiota in foregut (FG), midgut (MG), hindgut (HG). C: Each bar represents average relative abundance of each Familyof gut microbiota in foregut (FG), midgut (MG), hindgut (HG). The community bar plot shows the percentage abundance at different taxonomic levels, with species with relative abundance less than 1% represented by other species.


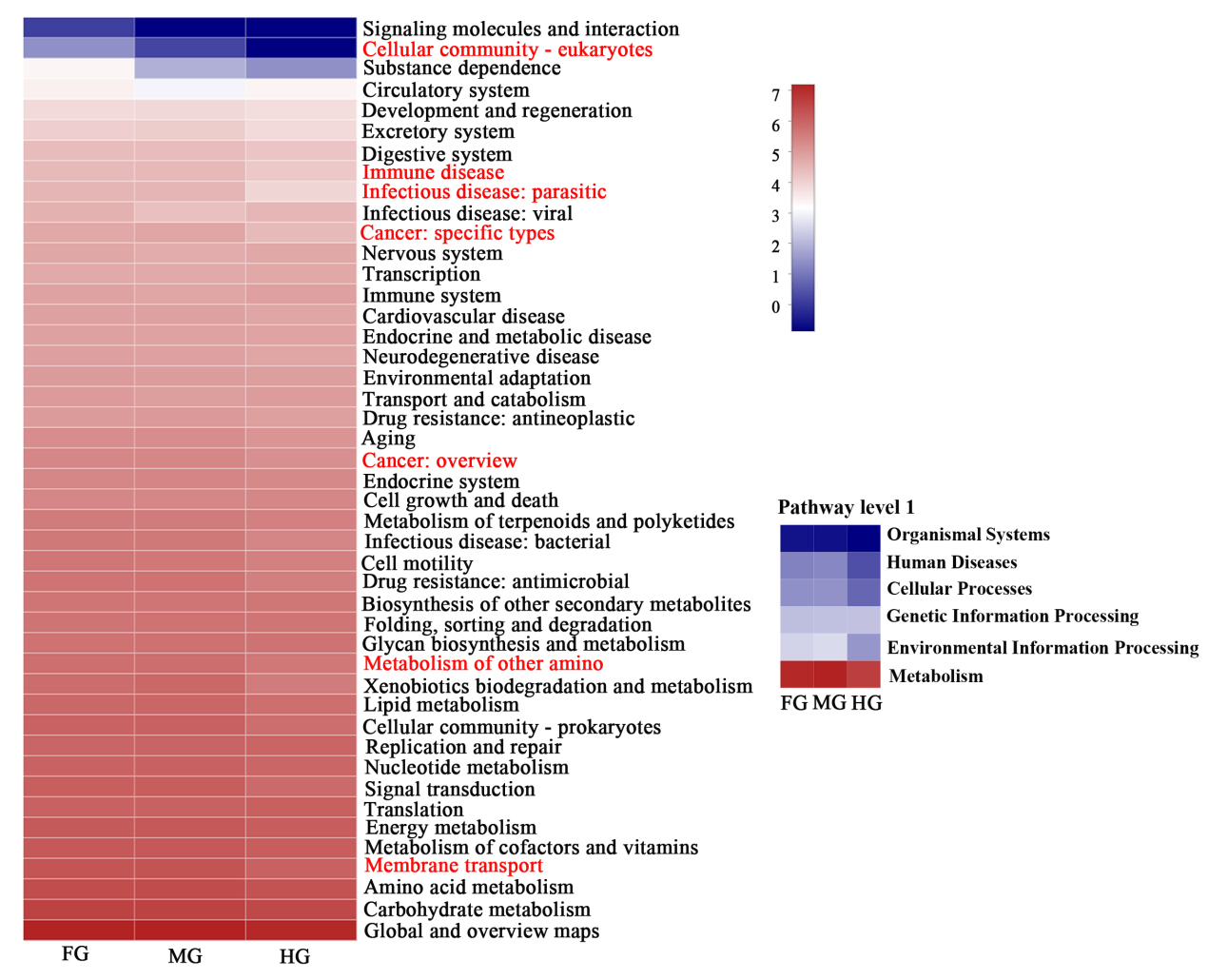


**Supplementary Figure 2** Heatmap host-gut microbiota functions of the final-instar larva of *P. xanthodes* at the Kyoto Encyclopedia of Genes and Genomes (KEGG) level 2.The one-way ANOVA analysis of variance was used to analyze the significant differences of species among foregut (FG), midgut (MG) and hindgut (HG). Red marks are pathways with significant differences in gut flora (*P* ＜ 0.05).

**Supplementary Table1** The number of microbial taxa identified from samples.

| Sample | Kindom | Phylum | Class | Order | Family | Genus | Species | ASV |
| --- | --- | --- | --- | --- | --- | --- | --- | --- |
| FG | 1 | 11 | 15 | 36 | 64 | 92 | 102 | 220 |
| MG | 1 | 12 | 16 | 33 | 54 | 67 | 78 | 159 |
| HG | 1 | 15 | 30 | 52 | 87 | 107 | 139 | 390 |

Gut microbial compositions at the different level of the final-instar larva. FG: foregut, MG: midgut , HG: hindgut.

**Supplemently Table 2** Comparison of microbiota relative abundance at the phylum level in the gut flora of the final-instar larva.

| Phylum | FG-mean(%) | FG-sd(%) | MG-mean(%) | MG-sd(%) | HG-mean(%) | HG-sd(%) | Statistic | *P*value | corrected pvalue |
| --- | --- | --- | --- | --- | --- | --- | --- | --- | --- |
| Proteobacteria | 51.38 | 35.98 | 65.07 | 23.24 | 16.78 | 19.42 | 3.288888889 | 0.1931 | 0.2446 |
| Firmicutes | 38.42 | 29.41 | 30.47 | 19.21 | 41.19 | 16.81 | 0.8 | 0.6703 | 0.6703 |
| Bacteroidota | 7.859 | 4.504 | 3.425 | 4.238 | 26.25 | 4.398 | 5.955555556 | 0.05091 | 0.09672 |
| Desulfobacterota | 0 | 0 | 0.2061 | 0.04181 | 5.689 | 0.9825 | 7.448275862 | 0.02413 | 0.09011 |
| Actinobacteriota | 2.163 | 1.999 | 0.2522 | 0.2265 | 2.126 | 2.146 | 4.355555556 | 0.1133 | 0.1656 |
| Fusobacteriota | 0 | 0 | 0.2761 | 0.2244 | 3.361 | 1.303 | 7.448275862 | 0.02413 | 0.09011 |
| Synergistota | 0 | 0 | 0.03865 | 0.04169 | 2.323 | 0.609 | 6.763636364 | 0.03399 | 0.09011 |
| Rs-K70_termite_group | 0 | 0 | 0.07915 | 0.08978 | 0.9148 | 0.08858 | 6.763636364 | 0.03399 | 0.09011 |
| unclassified_k__norank_  d__Bacteria | 0.001841 | 0.003188 | 0.09571 | 0.03918 | 0.3755 | 0.4048 | 6.543417367 | 0.03794 | 0.09011 |
| Elusimicrobiota | 0 | 0 | 0 | 0 | 0.4086 | 0.4543 | 7.623529412 | 0.02211 | 0.09011 |
| Planctomycetota | 0 | 0 | 0.0497 | 0.05973 | 0.254 | 0.05061 | 6.763636364 | 0.03399 | 0.09011 |
| Patescibacteria | 0.08835 | 0.06223 | 0.01472 | 0.0139 | 0.1012 | 0.05363 | 3.921568627 | 0.1407 | 0.191 |
| Spirochaetota | 0 | 0 | 0 | 0 | 0.1123 | 0.0355 | 7.623529412 | 0.02211 | 0.09011 |
| Deferribacterota | 0 | 0 | 0.02209 | 0.03826 | 0.07178 | 0.03448 | 4.586666667 | 0.1009 | 0.1656 |
| Bdellovibrionota | 0.06074 | 0.0958 | 0 | 0 | 0 | 0 | 4.5 | 0.1054 | 0.1656 |
| Verrucomicrobiota | 0.003681 | 0.006376 | 0 | 0 | 0.03681 | 0.0314 | 6.168350168 | 0.04577 | 0.09662 |
| Chloroflexi | 0.02025 | 0.03507 | 0 | 0 | 0 | 0 | 2 | 0.3679 | 0.3883 |
| Myxococcota | 0.005522 | 0.009564 | 0 | 0 | 0 | 0 | 2 | 0.3679 | 0.3883 |
| Deinococcota | 0.001841 | 0.003188 | 0 | 0 | 0 | 0 | 2 | 0.3679 | 0.3883 |

The Kruskal-Wallis H test was employed to compare the microbiota relative abundance of different gut flora at the phylum level in the final-instar larva across multiple groups. To account for potential false positives, False Discovery Rate (FDR) correction was applied. Post-hoc tests using Tukey-Kramer (0.95) were utilized to determine differences between multiple sample groups that demonstrated significant differences. "sd" refers to the standard deviation of the samples.

**Supplemently Table 3** Comparison of microbiota relative abundance at the genus level in the gut flora of the final-instar larva**.**

| Species name | FG-mean(%) | FG-sd(%) | MG-mean(%) | MG-sd(%) | HG-mean(%) | HG-sd(%) | Statistic | *P*value | corrected *p*value |
| --- | --- | --- | --- | --- | --- | --- | --- | --- | --- |
| Hafnia-Obesumbacterium | 38.29 | 36.46 | 42.49 | 37.44 | 0.254 | 0.2371 | 2.488888889 | 0.2881 | 0.3946 |
| Lactobacillus | 19.68 | 33.35 | 4.228 | 4.797 | 21.54 | 19.85 | 0.266666667 | 0.8752 | 0.8801 |
| Lactococcus | 13.32 | 14.1 | 20.63 | 14.86 | 4.966 | 5.765 | 3.2 | 0.2019 | 0.3279 |
| unclassified_f__Enterobacteriaceae | 3.93 | 3.833 | 8.77 | 8.056 | 12.18 | 19.43 | 0.8 | 0.6703 | 0.678 |
| Acinetobacter | 1.182 | 1.208 | 10.75 | 15.31 | 0.01657 | 0.01657 | 6.488888889 | 0.03899 | 0.1078 |
| Alistipes | 0 | 0 | 0.03129 | 0.008435 | 9.875 | 4.053 | 7.448275862 | 0.02413 | 0.09916 |
| Chryseobacterium | 4.651 | 1.579 | 2.864 | 4.602 | 0 | 0 | 5.793103448 | 0.05521 | 0.1396 |
| Enterococcus | 1.388 | 1.327 | 4.132 | 2.329 | 1.257 | 0.2473 | 4.355555556 | 0.1133 | 0.2005 |
| Dysgonomonas | 1.403 | 2.296 | 0.3424 | 0.3548 | 4.081 | 2.201 | 3.288888889 | 0.1931 | 0.3257 |
| Desulfovibrio | 0 | 0 | 0.127 | 0.04313 | 5.076 | 1.048 | 7.448275862 | 0.02413 | 0.09916 |
| Christensenellaceae_R-7_group | 0 | 0 | 0.04233 | 0.008435 | 4.804 | 1.433 | 7.448275862 | 0.02413 | 0.09916 |
| Weissella | 3.326 | 4.519 | 0.8209 | 1.304 | 0.03313 | 0.05738 | 1.915151515 | 0.3838 | 0.4093 |
| Macellibacteroides | 0 | 0 | 0.03129 | 0.04497 | 3.709 | 1.195 | 6.763636364 | 0.03399 | 0.09916 |
| unclassified_f__Tannerellaceae | 0.005522 | 0.009564 | 0.03865 | 0.0452 | 3.422 | 0.4688 | 6.16091954 | 0.04594 | 0.1214 |
| Fusobacterium | 0 | 0 | 0.2669 | 0.2198 | 3.175 | 1.237 | 7.448275862 | 0.02413 | 0.09916 |
| Pseudomonas | 0.5761 | 0.388 | 0.005522 | 0.009564 | 2.615 | 1.663 | 7.260504202 | 0.02651 | 0.09916 |
| Morganella | 1.327 | 1.153 | 1.403 | 1.608 | 0.2154 | 0.147 | 3.288888889 | 0.1931 | 0.3257 |
| norank_f__Ruminococcaceae | 0.005522 | 0.009564 | 0.07546 | 0.03374 | 2.512 | 1.44 | 7.260504202 | 0.02651 | 0.09916 |
| unclassified_o__Micrococcales | 0 | 0 | 0 | 0 | 1.874 | 2.094 | 7.623529412 | 0.02211 | 0.09916 |
| Aeromonas | 1.047 | 0.8952 | 0.5964 | 0.7536 | 0.04233 | 0.0686 | 3.466666667 | 0.1767 | 0.3066 |
| Ignatzschineria | 0 | 0 | 0.2724 | 0.2825 | 1.178 | 0.2191 | 7.448275862 | 0.02413 | 0.09916 |
| Syntrophus | 0 | 0 | 0 | 0 | 0.001841 | 0.003188 | 2 | 0.3679 | 0.3946 |
| Species name | FG-mean(%) | FG-sd(%) | MG-mean(%) | MG-sd(%) | HG-mean(%) | HG-sd(%) | Statistic | *P*value | corrected *p*value |
| Sphingobacterium | 1.419 | 1.46 | 0.02761 | 0.03074 | 0 | 0 | 6.763636364 | 0.03399 | 0.09916 |
| Candidatus_Tammella | 0 | 0 | 0.03865 | 0.04169 | 1.15 | 0.4941 | 6.763636364 | 0.03399 | 0.09916 |
| Anaerovorax | 0 | 0 | 0.04049 | 0.03878 | 1.143 | 0.2071 | 6.763636364 | 0.03399 | 0.09916 |
| unclassified_f__Synergistaceae | 0 | 0 | 0 | 0 | 1.172 | 0.2874 | 7.623529412 | 0.02211 | 0.09916 |
| norank_f__Rikenellaceae | 0 | 0 | 0 | 0 | 1.136 | 1.036 | 7.623529412 | 0.02211 | 0.09916 |
| Enterobacter | 0.7105 | 1.231 | 0.3681 | 0.6376 | 0 | 0 | 1.166666667 | 0.558 | 0.5709 |
| norank_f__norank_o__norank_c__norank_p__Rs-K70_termite_group | 0 | 0 | 0.07915 | 0.08978 | 0.9148 | 0.08858 | 6.763636364 | 0.03399 | 0.09916 |
| Bacteroides | 0 | 0 | 0.01288 | 0.02232 | 0.9682 | 0.5483 | 6.72 | 0.03474 | 0.09916 |
| Stenotrophomonas | 0.8909 | 1.127 | 0.04417 | 0.03865 | 0 | 0 | 6.763636364 | 0.03399 | 0.09916 |
| Vagococcus | 0.5872 | 0.6213 | 0.1749 | 0.1738 | 0.1049 | 0.1452 | 2.755555556 | 0.2521 | 0.3946 |
| unclassified_o__Lactobacillales | 0 | 0 | 0.02393 | 0.04144 | 0.8154 | 0.7101 | 3.231372549 | 0.1988 | 0.3257 |
| Candidatus_Soleaferrea | 0 | 0 | 0.06994 | 0.04598 | 0.7491 | 0.0564 | 7.448275862 | 0.02413 | 0.09916 |
| unclassified_o__Bacteroidales | 0 | 0 | 0 | 0 | 0.7381 | 0.8887 | 7.623529412 | 0.02211 | 0.09916 |
| Tyzzerella | 0 | 0 | 0.04602 | 0.04217 | 0.6663 | 0.1666 | 6.763636364 | 0.03399 | 0.09916 |
| Comamonas | 0.6516 | 0.8633 | 0.04602 | 0.06588 | 0 | 0 | 5.842424242 | 0.05387 | 0.1382 |
| Gordonia | 0.5982 | 0.842 | 0 | 0 | 0 | 0 | 7.623529412 | 0.02211 | 0.09916 |
| Ensifer | 0.5595 | 0.4915 | 0.02209 | 0.0253 | 0 | 0 | 6.763636364 | 0.03399 | 0.09916 |
| unclassified_k__norank_d__Bacteria | 0.001841 | 0.003188 | 0.09571 | 0.03918 | 0.3755 | 0.4048 | 6.543417367 | 0.03794 | 0.1066 |
| unclassified_f__Ruminococcaceae | 0 | 0 | 0.005522 | 0.009564 | 0.4454 | 0.1295 | 6.72 | 0.03474 | 0.09916 |
| Serratia | 0.335 | 0.5331 | 0.09019 | 0.1562 | 0.01841 | 0.03188 | 1.050505051 | 0.5914 | 0.6016 |
| BCf9-17_termite_group | 0 | 0 | 0.01472 | 0.0255 | 0.4086 | 0.3272 | 6.72 | 0.03474 | 0.09916 |
| norank_f__Paludibacteraceae | 0 | 0 | 0 | 0 | 0.3939 | 0.4648 | 7.623529412 | 0.02211 | 0.09916 |
| Microbacterium | 0.3663 | 0.2488 | 0 | 0 | 0 | 0 | 7.623529412 | 0.02211 | 0.09916 |
| Species name | FG-mean(%) | FG-sd(%) | MG-mean(%) | MG-sd(%) | HG-mean(%) | HG-sd(%) | Statistic | *P*value | corrected *p*value |
| Ochrobactrum | 0.3424 | 0.2431 | 0.02209 | 0.03826 | 0 | 0 | 6.168350168 | 0.04577 | 0.1214 |
| Endomicrobium | 0 | 0 | 0 | 0 | 0.3608 | 0.491 | 7.623529412 | 0.02211 | 0.09916 |
| unclassified_f__Desulfovibrionaceae | 0 | 0 | 0.04786 | 0.008435 | 0.2982 | 0.02407 | 7.448275862 | 0.02413 | 0.09916 |
| Butyricimonas | 0 | 0 | 0.005522 | 0.009564 | 0.3368 | 0.05844 | 6.72 | 0.03474 | 0.09916 |
| Empedobacter | 0.2761 | 0.4782 | 0.04602 | 0.0797 | 0.01104 | 0.01913 | 0.125490196 | 0.9392 | 0.9392 |
| Corynebacterium | 0.116 | 0.2008 | 0.2006 | 0.2202 | 0.01288 | 0.02232 | 1.386666667 | 0.4999 | 0.5298 |
| norank_f__norank_o__norank_c__vadinHA49 | 0 | 0 | 0.0497 | 0.05973 | 0.254 | 0.05061 | 6.763636364 | 0.03399 | 0.09916 |
| Rhodococcus | 0.2595 | 0.3595 | 0.03681 | 0.0139 | 0 | 0 | 6.16091954 | 0.04594 | 0.1214 |
| Acholeplasma | 0.003681 | 0.006376 | 0.01472 | 0.0255 | 0.2522 | 0.1135 | 5.915151515 | 0.05194 | 0.1352 |
| Leucobacter | 0.2595 | 0.2547 | 0 | 0 | 0.009203 | 0.01594 | 5.546666667 | 0.06245 | 0.1557 |
| unclassified_o__Enterobacterales | 0.05706 | 0.09883 | 0.1325 | 0.1023 | 0.0681 | 0.06634 | 1.195402299 | 0.5501 | 0.5709 |
| Shinella | 0.2558 | 0.3519 | 0 | 0 | 0 | 0 | 4.5 | 0.1054 | 0.1884 |
| unclassified_f__Rikenellaceae | 0 | 0 | 0 | 0 | 0.2466 | 0.1597 | 7.623529412 | 0.02211 | 0.09916 |
| unclassified_f__Micrococcaceae | 0.2282 | 0.3441 | 0.01472 | 0.0255 | 0 | 0 | 3.231372549 | 0.1988 | 0.3257 |
| unclassified_f__Christensenellaceae | 0 | 0 | 0 | 0 | 0.2374 | 0.2056 | 7.623529412 | 0.02211 | 0.09916 |
| unclassified_f__Anaerovoracaceae | 0 | 0 | 0.009203 | 0.01594 | 0.2006 | 0.1255 | 6.72 | 0.03474 | 0.09916 |
| norank_f__Marinilabiliaceae | 0 | 0 | 0 | 0 | 0.2098 | 0.1141 | 7.623529412 | 0.02211 | 0.09916 |
| Rikenella | 0 | 0 | 0 | 0 | 0.2006 | 0.338 | 4.5 | 0.1054 | 0.1884 |
| Sebaldella | 0 | 0 | 0.009203 | 0.01594 | 0.1859 | 0.07497 | 6.72 | 0.03474 | 0.09916 |
| unclassified_o__Oscillospirales | 0 | 0 | 0 | 0 | 0.1859 | 0.1274 | 7.623529412 | 0.02211 | 0.09916 |
| unclassified_o__Desulfobacterales | 0 | 0 | 0.01841 | 0.01687 | 0.162 | 0.05131 | 6.763636364 | 0.03399 | 0.09916 |
| Koukoulia | 0.162 | 0.2805 | 0.01472 | 0.0255 | 0 | 0 | 1.166666667 | 0.558 | 0.5709 |
| unclassified_c__Bacteroidia | 0 | 0 | 0.01104 | 0.01913 | 0.1583 | 0.111 | 6.72 | 0.03474 | 0.09916 |
| Anaerofustis | 0 | 0 | 0 | 0 | 0.001841 | 0.003188 | 2 | 0.3679 | 0.3946 |
| Species name | FG-mean(%) | FG-sd(%) | MG-mean(%) | MG-sd(%) | HG-mean(%) | HG-sd(%) | Statistic | *P*value | corrected *p*value |
| norank_f__Eubacterium_coprostanoligenes_group | 0 | 0 | 0 | 0 | 0.1693 | 0.03328 | 7.623529412 | 0.02211 | 0.09916 |
| norank_f__Moraxellaceae | 0.162 | 0.2805 | 0 | 0 | 0 | 0 | 2 | 0.3679 | 0.3946 |
| Rs-D38_termite_group | 0 | 0 | 0 | 0 | 0.162 | 0.0628 | 7.623529412 | 0.02211 | 0.09916 |
| norank_f__Acidaminococcaceae | 0 | 0 | 0 | 0 | 0.1565 | 0.05131 | 7.623529412 | 0.02211 | 0.09916 |
| unclassified_o__Coriobacteriales | 0 | 0 | 0 | 0 | 0.1528 | 0.03677 | 7.623529412 | 0.02211 | 0.09916 |
| Bosea | 0.1491 | 0.1613 | 0 | 0 | 0 | 0 | 4.5 | 0.1054 | 0.1884 |
| Breznakia | 0.04417 | 0.04417 | 0.001841 | 0.003188 | 0.08467 | 0.07989 | 3.779710145 | 0.1511 | 0.2648 |
| Tsukamurella | 0.1252 | 0.2168 | 0 | 0 | 0 | 0 | 2 | 0.3679 | 0.3946 |
| Carnobacterium | 0.04233 | 0.07332 | 0.08283 | 0.1435 | 0 | 0 | 1.166666667 | 0.558 | 0.5709 |
| Delftia | 0.1196 | 0.09821 | 0.005522 | 0.009564 | 0 | 0 | 6.72 | 0.03474 | 0.09916 |
| TC1 | 0 | 0 | 0 | 0 | 0.1233 | 0.06588 | 7.623529412 | 0.02211 | 0.09916 |
| norank_f__norank_o__norank_c__MVP-15 | 0 | 0 | 0 | 0 | 0.1123 | 0.0355 | 7.623529412 | 0.02211 | 0.09916 |
| unclassified_f__Rhizobiaceae | 0.1086 | 0.09441 | 0 | 0 | 0 | 0 | 4.5 | 0.1054 | 0.1884 |
| unclassified_c__Clostridia | 0 | 0 | 0.003681 | 0.006376 | 0.1031 | 0.07332 | 6.787878788 | 0.03358 | 0.09916 |
| norank_f__norank_o__Rhodospirillales | 0 | 0 | 0 | 0 | 0.1031 | 0.04949 | 7.623529412 | 0.02211 | 0.09916 |
| norank_f__Christensenellaceae | 0 | 0 | 0.003681 | 0.006376 | 0.09939 | 0.05267 | 6.72 | 0.03474 | 0.09916 |
| Mucispirillum | 0 | 0 | 0.02209 | 0.03826 | 0.07178 | 0.03448 | 4.586666667 | 0.1009 | 0.1884 |
| Sphingopyxis | 0.07731 | 0.08678 | 0.003681 | 0.006376 | 0 | 0 | 3.231372549 | 0.1988 | 0.3257 |
| norank_f__norank_o__Desulfobacterales | 0 | 0 | 0.01288 | 0.01149 | 0.0681 | 0.05131 | 6.763636364 | 0.03399 | 0.09916 |
| unclassified_o__Lachnospirales | 0 | 0 | 0 | 0 | 0.07915 | 0.01594 | 7.714285714 | 0.02113 | 0.09916 |
| Legionella | 0.001841 | 0.003188 | 0 | 0 | 0 | 0 | 2 | 0.3679 | 0.3946 |
| Truepera | 0.001841 | 0.003188 | 0 | 0 | 0 | 0 | 2 | 0.3679 | 0.3946 |
| Achromobacter | 0.001841 | 0.003188 | 0 | 0 | 0 | 0 | 2 | 0.3679 | 0.3946 |
| Nocardioides | 0.001841 | 0.003188 | 0 | 0 | 0 | 0 | 2 | 0.3679 | 0.3946 |
| Species name | FG-mean(%) | FG-sd(%) | MG-mean(%) | MG-sd(%) | HG-mean(%) | HG-sd(%) | Statistic | *P*value | corrected *p*value |
| Nitrosomonas | 0 | 0 | 0.01472 | 0.01275 | 0.06258 | 0.01939 | 6.825688073 | 0.03295 | 0.09916 |
| unclassified_c__Gammaproteobacteria | 0.06994 | 0.1211 | 0 | 0 | 0 | 0 | 2 | 0.3679 | 0.3946 |
| Pseudochrobactrum | 0.0681 | 0.04144 | 0 | 0 | 0 | 0 | 7.714285714 | 0.02113 | 0.09916 |
| CAG-352 | 0 | 0 | 0 | 0 | 0.0681 | 0.118 | 2 | 0.3679 | 0.3946 |
| Spiroplasma | 0 | 0 | 0.0589 | 0.102 | 0.005522 | 0.009564 | 1.166666667 | 0.558 | 0.5709 |
| Candidatus_Saccharimonas | 0 | 0 | 0.005522 | 0.009564 | 0.05706 | 0.05586 | 6.72 | 0.03474 | 0.09916 |
| Brevibacterium | 0.06074 | 0.0958 | 0 | 0 | 0 | 0 | 4.5 | 0.1054 | 0.1884 |
| Desulfobulbus | 0 | 0 | 0 | 0 | 0.06074 | 0.09124 | 4.5 | 0.1054 | 0.1884 |
| unclassified_p__Firmicutes | 0 | 0 | 0 | 0 | 0.05706 | 0.08944 | 4.5 | 0.1054 | 0.1884 |
| UCG-009 | 0 | 0 | 0 | 0 | 0.05522 | 0.01913 | 7.714285714 | 0.02113 | 0.09916 |
| Peredibacter | 0.05338 | 0.09245 | 0 | 0 | 0 | 0 | 2 | 0.3679 | 0.3946 |
| Odoribacter | 0 | 0 | 0 | 0 | 0.05338 | 0.03878 | 7.623529412 | 0.02211 | 0.09916 |
| Flavobacterium | 0.04602 | 0.05363 | 0 | 0 | 0.005522 | 0.009564 | 3.231372549 | 0.1988 | 0.3257 |
| Mucinivorans | 0 | 0 | 0 | 0 | 0.05154 | 0.05559 | 4.5 | 0.1054 | 0.1884 |
| norank_f__CR-115 | 0 | 0 | 0 | 0 | 0.05154 | 0.0498 | 4.5 | 0.1054 | 0.1884 |
| Mycobacterium | 0.05154 | 0.0564 | 0 | 0 | 0 | 0 | 7.623529412 | 0.02211 | 0.09916 |
| Proteus | 0.03129 | 0.0407 | 0.01841 | 0.03188 | 0 | 0 | 2.541176471 | 0.2807 | 0.3946 |
| Elusimicrobium | 0 | 0 | 0 | 0 | 0.04786 | 0.03677 | 7.623529412 | 0.02211 | 0.09916 |
| norank_f__norank_o__norank_c__  BRH-c20a | 0 | 0 | 0 | 0 | 0.04602 | 0.04289 | 7.623529412 | 0.02211 | 0.09916 |
| norank_f__UCG-010 | 0 | 0 | 0 | 0 | 0.04602 | 0.008435 | 7.623529412 | 0.02211 | 0.09916 |
| TM7a | 0.04233 | 0.07332 | 0 | 0 | 0 | 0 | 2 | 0.3679 | 0.3946 |
| Raoultibacter | 0 | 0 | 0 | 0 | 0.03865 | 0.005522 | 7.623529412 | 0.02211 | 0.09916 |
| Bdellovibrio | 0.003681 | 0.006376 | 0 | 0 | 0 | 0 | 2 | 0.3679 | 0.3946 |
| Species name | FG-mean(%) | FG-sd(%) | MG-mean(%) | MG-sd(%) | HG-mean(%) | HG-sd(%) | Statistic | *P*value | corrected *p*value |
| Cavicella | 0.03681 | 0.06376 | 0 | 0 | 0 | 0 | 2 | 0.3679 | 0.3946 |
| unclassified_f__Rhodobacteraceae | 0.03497 | 0.06057 | 0 | 0 | 0 | 0 | 2 | 0.3679 | 0.3946 |
| norank_f__Coriobacteriales_Incertae_Sedis | 0 | 0 | 0 | 0 | 0.03497 | 0.03918 | 4.5 | 0.1054 | 0.1884 |
| Akkermansia | 0 | 0 | 0 | 0 | 0.03313 | 0.03621 | 4.5 | 0.1054 | 0.1884 |
| norank_f__norank_o__Candidatus_  Pacebacteria | 0 | 0 | 0.009203 | 0.01594 | 0.02209 | 0.01991 | 2.888888889 | 0.2359 | 0.3795 |
| norank_f__M2PB4-65_termite_  group | 0 | 0 | 0 | 0 | 0.03129 | 0.01594 | 7.714285714 | 0.02113 | 0.09916 |
| norank_f__norank_o__Saccharimonadales | 0.03129 | 0.0542 | 0 | 0 | 0 | 0 | 2 | 0.3679 | 0.3946 |
| unclassified_f__Lachnospiraceae | 0 | 0 | 0 | 0 | 0.02945 | 0.05101 | 2 | 0.3679 | 0.3946 |
| Dietzia | 0.02761 | 0.04782 | 0 | 0 | 0 | 0 | 2 | 0.3679 | 0.3946 |
| Brachybacterium | 0.02761 | 0.04782 | 0 | 0 | 0 | 0 | 2 | 0.3679 | 0.3946 |
| norank_f__Peptococcaceae | 0 | 0 | 0 | 0 | 0.02577 | 0.0249 | 4.5 | 0.1054 | 0.1884 |
| norank_f__norank_o__RF39 | 0 | 0 | 0 | 0 | 0.02393 | 0.01775 | 7.623529412 | 0.02211 | 0.09916 |
| norank_f__norank_o__norank_c__  norank_p__Firmicutes | 0 | 0 | 0 | 0 | 0.02393 | 0.02232 | 4.5 | 0.1054 | 0.1884 |
| Desulfobotulus | 0 | 0 | 0 | 0 | 0.02209 | 0.01913 | 4.571428571 | 0.1017 | 0.1884 |
| Ruminococcus | 0 | 0 | 0 | 0 | 0.02209 | 0.03826 | 2 | 0.3679 | 0.3946 |
| norank_f__norank_o__JGI_0000069-P22 | 0 | 0 | 0 | 0 | 0.02209 | 0.03826 | 2 | 0.3679 | 0.3946 |
| Devosia | 0.02209 | 0.02209 | 0 | 0 | 0 | 0 | 4.5 | 0.1054 | 0.1884 |
| Gemmobacter | 0.02209 | 0.02209 | 0 | 0 | 0 | 0 | 4.5 | 0.1054 | 0.1884 |
| unclassified_f__Beijerinckiaceae | 0.003681 | 0.006376 | 0 | 0 | 0 | 0 | 2 | 0.3679 | 0.3946 |
| Roseimicrobium | 0.003681 | 0.006376 | 0 | 0 | 0 | 0 | 2 | 0.3679 | 0.3946 |
| Niabella | 0.003681 | 0.006376 | 0 | 0 | 0 | 0 | 2 | 0.3679 | 0.3946 |
| Species name | FG-mean(%) | FG-sd(%) | MG-mean(%) | MG-sd(%) | HG-mean(%) | HG-sd(%) | Statistic | *P*value | corrected *p*value |
| Pseudoxanthomonas | 0.02025 | 0.01775 | 0 | 0 | 0 | 0 | 4.5 | 0.1054 | 0.1884 |
| norank_f__JG30-KF-CM45 | 0.02025 | 0.03507 | 0 | 0 | 0 | 0 | 2 | 0.3679 | 0.3946 |
| Paracoccus | 0.02025 | 0.03507 | 0 | 0 | 0 | 0 | 2 | 0.3679 | 0.3946 |
| unclassified_f__Rhodocyclaceae | 0 | 0 | 0 | 0 | 0.01841 | 0.02724 | 4.5 | 0.1054 | 0.1884 |
| unclassified_f__Oscillospiraceae | 0 | 0 | 0 | 0 | 0.01841 | 0.01939 | 4.5 | 0.1054 | 0.1884 |
| Pedobacter | 0.01657 | 0.01991 | 0 | 0 | 0 | 0 | 4.5 | 0.1054 | 0.1884 |
| unclassified_f__Comamonadaceae | 0.01472 | 0.0255 | 0 | 0 | 0 | 0 | 2 | 0.3679 | 0.3946 |
| Allorhizobium-Neorhizobium-  Pararhizobium-Rhizobium | 0.01472 | 0.0255 | 0 | 0 | 0 | 0 | 2 | 0.3679 | 0.3946 |
| unclassified_f__Intrasporangiaceae | 0.01472 | 0.0255 | 0 | 0 | 0 | 0 | 2 | 0.3679 | 0.3946 |
| Staphylococcus | 0.01472 | 0.0255 | 0 | 0 | 0 | 0 | 2 | 0.3679 | 0.3946 |
| Elizabethkingia | 0.01472 | 0.0255 | 0 | 0 | 0 | 0 | 2 | 0.3679 | 0.3946 |
| Taibaiella | 0.01472 | 0.01275 | 0 | 0 | 0 | 0 | 4.571428571 | 0.1017 | 0.1884 |
| unclassified_o__Saccharimonadales | 0.01472 | 0.0139 | 0 | 0 | 0 | 0 | 4.5 | 0.1054 | 0.1884 |
| norank_f__norank_o__Clostridia_  UCG-014 | 0 | 0 | 0 | 0 | 0.01288 | 0.0139 | 4.5 | 0.1054 | 0.1884 |
| Aquabacterium | 0.01288 | 0.01149 | 0 | 0 | 0 | 0 | 4.5 | 0.1054 | 0.1884 |
| Papillibacter | 0 | 0 | 0 | 0 | 0.01288 | 0.02232 | 2 | 0.3679 | 0.3946 |
| Timonella | 0.01104 | 0.01913 | 0 | 0 | 0 | 0 | 2 | 0.3679 | 0.3946 |
| unclassified_f__Yersiniaceae | 0.01104 | 0.01913 | 0 | 0 | 0 | 0 | 2 | 0.3679 | 0.3946 |
| Brevundimonas | 0.01104 | 0.01913 | 0 | 0 | 0 | 0 | 2 | 0.3679 | 0.3946 |
| Candidatus_Berkiella | 0.009203 | 0.01594 | 0 | 0 | 0 | 0 | 2 | 0.3679 | 0.3946 |
| Dyadobacter | 0.009203 | 0.008435 | 0 | 0 | 0 | 0 | 4.5 | 0.1054 | 0.1884 |
| Iamia | 0.009203 | 0.01594 | 0 | 0 | 0 | 0 | 2 | 0.3679 | 0.3946 |
| Species name | FG-mean(%) | FG-sd(%) | MG-mean(%) | MG-sd(%) | HG-mean(%) | HG-sd(%) | Statistic | *P*value | corrected *p*value |
| unclassified_o__Burkholderiales | 0 | 0 | 0 | 0 | 0.009203 | 0.008435 | 4.5 | 0.1054 | 0.1884 |
| Advenella | 0.007362 | 0.01275 | 0 | 0 | 0 | 0 | 2 | 0.3679 | 0.3946 |
| Thermomonas | 0.007362 | 0.01275 | 0 | 0 | 0 | 0 | 2 | 0.3679 | 0.3946 |
| Monoglobus | 0 | 0 | 0 | 0 | 0.007362 | 0.01275 | 2 | 0.3679 | 0.3946 |
| Kaistia | 0.005522 | 0.009564 | 0 | 0 | 0 | 0 | 2 | 0.3679 | 0.3946 |
| unclassified_f__Xanthobacteraceae | 0.005522 | 0.009564 | 0 | 0 | 0 | 0 | 2 | 0.3679 | 0.3946 |
| Aeromicrobium | 0.005522 | 0.009564 | 0 | 0 | 0 | 0 | 2 | 0.3679 | 0.3946 |
| Caulobacter | 0.005522 | 0.009564 | 0 | 0 | 0 | 0 | 2 | 0.3679 | 0.3946 |
| unclassified_f__Myxococcaceae | 0.005522 | 0.009564 | 0 | 0 | 0 | 0 | 2 | 0.3679 | 0.3946 |
| unclassified_f__Oxalobacteraceae | 0 | 0 | 0.003681 | 0.006376 | 0.001841 | 0.003188 | 1.166666667 | 0.558 | 0.5709 |
| Sphingomonas | 0.005522 | 0.009564 | 0 | 0 | 0 | 0 | 2 | 0.3679 | 0.3946 |
| Roseburia | 0 | 0 | 0 | 0 | 0.005522 | 0.009564 | 2 | 0.3679 | 0.3946 |
| norank_f__norank_o__Veillonellales-Selenomonadales | 0 | 0 | 0 | 0 | 0.003681 | 0.006376 | 2 | 0.3679 | 0.3946 |
| norank_f__norank_o__OPB41 | 0 | 0 | 0 | 0 | 0.003681 | 0.006376 | 2 | 0.3679 | 0.3946 |
| norank_f__norank_o__Clostridia_  vadinBB60_group | 0 | 0 | 0 | 0 | 0.003681 | 0.003188 | 4.571428571 | 0.1017 | 0.1884 |
| Leuconostoc | 0 | 0 | 0.003681 | 0.006376 | 0 | 0 | 2 | 0.3679 | 0.3946 |
| norank_f__Silvanigrellaceae | 0.003681 | 0.006376 | 0 | 0 | 0 | 0 | 2 | 0.3679 | 0.3946 |
| norank_f__Victivallaceae | 0 | 0 | 0 | 0 | 0.003681 | 0.006376 | 2 | 0.3679 | 0.3946 |

The Kruskal-Wallis H test was employed to compare the microbiota relative abundance of different gut flora at the genus level in the final-instar larva across multiple groups. To account for potential false positives, False Discovery Rate (FDR) correction was applied. Post-hoc tests using Tukey-Kramer (0.95) were utilized to determine differences between multiple sample groups that demonstrated significant differences.

**Supplementary Table 4** Enriched KEGG pathways (level 1, 2 and 3) according to the gut microbiota of the final-instar larva.

| Pathway level1 | Pathway level2 | Pathway level3 | Description | FG | MG | HG |
| --- | --- | --- | --- | --- | --- | --- |
|  |  |  |  | Relative abundance | | |
| Metabolism | Global and overview maps | ko01100 | Metabolic pathways | 7015124.89 | 7411913.41 | 5707448.77 |
| Metabolism | Global and overview maps | ko01110 | Biosynthesis of secondary metabolites | 2968949.41 | 3092498.75 | 2699973.48 |
| Metabolism | Global and overview maps | ko01120 | Microbial metabolism in diverse environments | 2047694.62 | 2244724.77 | 1427625.24 |
| Metabolism | Global and overview maps | ko01230 | Biosynthesis of amino acids | 1153039.01 | 1217842.56 | 1142767.24 |
| Environmental Information Processing | Membrane transport | ko02010 | ABC transporters | 1167485.31 | 1211917.59 | 731304.95 |
| Metabolism | Global and overview maps | ko01200 | Carbon metabolism | 1024965.04 | 1059036.89 | 901991.41 |
| Environmental Information Processing | Signal transduction | ko02020 | Two-component system | 946692.02 | 1021585.48 | 555312.21 |
| Genetic Information Processing | Translation | ko03010 | Ribosome | 712141.16 | 687992.13 | 795216.57 |
| Metabolism | Nucleotide metabolism | ko00230 | Purine metabolism | 634840.37 | 648106.62 | 516094.44 |
| Cellular Processes | Cellular community - prokaryotes | ko02024 | Quorum sensing | 523422.23 | 531446.31 | 410531.7 |
| Metabolism | Carbohydrate metabolism | ko00010 | Glycolysis / Gluconeogenesis | 454626.02 | 460904.43 | 400909.69 |
| Metabolism | Carbohydrate metabolism | ko00620 | Pyruvate metabolism | 426651.44 | 459450.09 | 346416.21 |
| Metabolism | Carbohydrate metabolism | ko00520 | Amino sugar and nucleotide sugar metabolism | 424357.2 | 409013.8 | 394152.94 |
| Metabolism | Nucleotide metabolism | ko00240 | Pyrimidine metabolism | 399007.69 | 416394.6 | 372819.08 |
| Metabolism | Carbohydrate metabolism | ko00500 | Starch and sucrose metabolism | 401456.2 | 420744.64 | 303408.56 |
| Metabolism | Energy metabolism | ko00190 | Oxidative phosphorylation | 352910.08 | 370278.44 | 295861.72 |
| Metabolism | Amino acid metabolism | ko00260 | Glycine, serine and threonine metabolism | 342999.05 | 339176.74 | 296730.42 |
| Environmental Information Processing | Membrane transport | ko02060 | Phosphotransferase system (PTS) | 385947.16 | 400075.68 | 187934.62 |
| Pathway level1 | Pathway level2 | Pathway level3 | Description | FG | MG | HG |
|  |  |  |  | Relative abundance | | |
| Genetic Information Processing | Translation | ko00970 | Aminoacyl-tRNA biosynthesis | 300616.33 | 312830.16 | 333602.41 |
| Metabolism | Amino acid metabolism | ko00270 | Cysteine and methionine metabolism | 317533.85 | 346288.62 | 279724.14 |
| Metabolism | Carbohydrate metabolism | ko00030 | Pentose phosphate pathway | 324924.48 | 339522.53 | 228243.81 |
| Metabolism | Energy metabolism | ko00720 | Carbon fixation pathways in prokaryotes | 273475.49 | 301592.09 | 294957.55 |
| Metabolism | Amino acid metabolism | ko00250 | Alanine, aspartate and glutamate metabolism | 291017.71 | 293722.78 | 272584.21 |
| Metabolism | Energy metabolism | ko00680 | Methane metabolism | 294324.46 | 294511.46 | 266818.62 |
| Genetic Information Processing | Replication and repair | ko03440 | Homologous recombination | 274542.87 | 288898.88 | 265228.57 |
| Metabolism | Carbohydrate metabolism | ko00630 | Glyoxylate and dicarboxylate metabolism | 282629.57 | 283543.94 | 233934.94 |
| Metabolism | Glycan biosynthesis and metabolism | ko00550 | Peptidoglycan biosynthesis | 256595.95 | 284673.36 | 247138.65 |
| Metabolism | Carbohydrate metabolism | ko00640 | Propanoate metabolism | 282663.66 | 314560.34 | 183837.15 |
| Environmental Information Processing | Membrane transport | ko03070 | Bacterial secretion system | 268989.99 | 310663.8 | 193491.63 |
| Cellular Processes | Cell motility | ko02040 | Flagellar assembly | 310512.07 | 288170.99 | 170686.12 |
| Metabolism | Carbohydrate metabolism | ko00650 | Butanoate metabolism | 264728.25 | 293466.19 | 195472.28 |
| Metabolism | Carbohydrate metabolism | ko00051 | Fructose and mannose metabolism | 263138.48 | 250472.79 | 235256.81 |
| Genetic Information Processing | Replication and repair | ko03430 | Mismatch repair | 230178.75 | 244521.52 | 228890.39 |
| Metabolism | Metabolism of cofactors and vitamins | ko00860 | Porphyrin and chlorophyll metabolism | 248603.23 | 251073.14 | 202290.42 |
| Metabolism | Global and overview maps | ko01212 | Fatty acid metabolism | 242861.37 | 266027.24 | 182643.63 |
| Metabolism | Global and overview maps | ko01210 | 2-Oxocarboxylic acid metabolism | 217368.09 | 239154.13 | 231428.88 |
| Metabolism | Carbohydrate metabolism | ko00052 | Galactose metabolism | 244998.51 | 243510.59 | 186543.52 |
| Metabolism | Carbohydrate metabolism | ko00020 | Citrate cycle (TCA cycle) | 225805.2 | 243427.53 | 204068.94 |
| Metabolism | Amino acid metabolism | ko00400 | Phenylalanine, tyrosine and tryptophan biosynthesis | 206430.2 | 217919.26 | 206188.01 |
| Pathway level1 | Pathway level2 | Pathway level3 | Description | FG | MG | HG |
|  |  |  |  | Relative abundance | | |
| Organismal Systems | Immune system | ko04611 | Platelet activation | 0.67 | 0.033 | 0.033 |
| Organismal Systems | Endocrine system | ko04913 | Ovarian steroidogenesis | 0.83 | 0.033 | 0.033 |
| Genetic Information Processing | Replication and repair | ko03030 | DNA replication | 201863.32 | 200722.96 | 204120.02 |
| Genetic Information Processing | Folding, sorting and degradation | ko03060 | Protein export | 194136.28 | 210369.74 | 194477.58 |
| Metabolism | Lipid metabolism | ko00564 | Glycerophospholipid metabolism | 211461.85 | 231682.08 | 150022.78 |
| Human Diseases | Drug resistance: antimicrobial | ko01501 | beta-Lactam resistance | 210053.83 | 231481.57 | 131341.71 |
| Metabolism | Energy metabolism | ko00920 | Sulfur metabolism | 202033.62 | 253661.93 | 112607.63 |
| Human Diseases | Drug resistance: antimicrobial | ko01503 | Cationic antimicrobial peptide (CAMP) resistance | 217765.37 | 237144.78 | 105100.12 |
| Metabolism | Metabolism of cofactors and vitamins | ko00770 | Pantothenate and CoA biosynthesis | 185621.41 | 187198.58 | 174201.76 |
| Metabolism | Metabolism of cofactors and vitamins | ko00790 | Folate biosynthesis | 189056.15 | 210225.01 | 141224.69 |
| Metabolism | Lipid metabolism | ko00061 | Fatty acid biosynthesis | 184635.9 | 192791.73 | 160152.31 |
| Cellular Processes | Cellular community - prokaryotes | ko02026 | Biofilm formation - Escherichia coli | 213095.3 | 230911.45 | 93305.15 |
| Genetic Information Processing | Folding, sorting and degradation | ko03018 | RNA degradation | 173933.38 | 177843.75 | 179418.23 |
| Metabolism | Metabolism of cofactors and vitamins | ko00760 | Nicotinate and nicotinamide metabolism | 184908.66 | 196929.38 | 148736.11 |
| Metabolism | Glycan biosynthesis and metabolism | ko00540 | Lipopolysaccharide biosynthesis | 183406.68 | 203330.77 | 134427.3 |
| Metabolism | Amino acid metabolism | ko00300 | Lysine biosynthesis | 153398.42 | 172804.36 | 157022.77 |
| Metabolism | Energy metabolism | ko00710 | Carbon fixation in photosynthetic organisms | 158661.31 | 173836.45 | 148069.78 |
| Metabolism | Metabolism of terpenoids and polyketides | ko00900 | Terpenoid backbone biosynthesis | 153296.3 | 161270.18 | 156609.18 |
| Metabolism | Metabolism of cofactors and vitamins | ko00670 | One carbon pool by folate | 151166 | 151289.08 | 160240.07 |
| Metabolism | Amino acid metabolism | ko00220 | Arginine biosynthesis | 148358.65 | 164342.77 | 139209.65 |
| Metabolism | Metabolism of cofactors and vitamins | ko00730 | Thiamine metabolism | 150128.14 | 143398.6 | 154000.16 |
| Pathway level1 | Pathway level2 | Pathway level3 | Description | FG | MG | HG |
|  |  |  |  | Relative abundance | | |
| Metabolism | Amino acid metabolism | ko00330 | Arginine and proline metabolism | 169220.3 | 153871.94 | 115907.68 |
| Metabolism | Lipid metabolism | ko00561 | Glycerolipid metabolism | 160714.13 | 157483.35 | 121294.11 |
| Cellular Processes | Cell motility | ko04810 | Regulation of actin cytoskeleton | 0.67 | 0.033 | 0.033 |
| Genetic Information Processing | Replication and repair | ko03410 | Base excision repair | 153026.48 | 142380.23 | 137938.19 |
| Cellular Processes | Cell growth and death | ko04112 | Cell cycle - Caulobacter | 136277.1 | 140853.9 | 144107.65 |
| Cellular Processes | Cell motility | ko02030 | Bacterial chemotaxis | 147462.66 | 134970.09 | 137559.18 |
| Metabolism | Amino acid metabolism | ko00290 | Valine, leucine and isoleucine biosynthesis | 141106.75 | 145451.58 | 131347.81 |
| Metabolism | Metabolism of other amino acids | ko00480 | Glutathione metabolism | 157225.09 | 168915.77 | 84556.25 |
| Metabolism | Metabolism of cofactors and vitamins | ko00780 | Biotin metabolism | 137635.36 | 155547.23 | 114123.9 |
| Metabolism | Amino acid metabolism | ko00280 | Valine, leucine and isoleucine degradation | 151580.02 | 153429.83 | 99138.99 |
| Cellular Processes | Cellular community - prokaryotes | ko05111 | Biofilm formation - Vibrio cholerae | 138651.21 | 158514.82 | 103656.16 |
| Metabolism | Xenobiotics biodegradation and metabolism | ko00983 | Drug metabolism - other enzymes | 137599.47 | 146463.77 | 109597.68 |
| Metabolism | Carbohydrate metabolism | ko00040 | Pentose and glucuronate interconversions | 149294.79 | 154337.05 | 86255.73 |
| Metabolism | Metabolism of other amino acids | ko00450 | Selenocompound metabolism | 142338.45 | 149754.13 | 97433.13 |
| Metabolism | Amino acid metabolism | ko00360 | Phenylalanine metabolism | 149048.79 | 171857.12 | 67559.43 |
| Metabolism | Metabolism of cofactors and vitamins | ko00130 | Ubiquinone and other terpenoid-quinone biosynthesis | 141695.98 | 159271.83 | 81799.38 |
| Metabolism | Amino acid metabolism | ko00310 | Lysine degradation | 154775.94 | 160749.48 | 63428.56 |
| Metabolism | Energy metabolism | ko00910 | Nitrogen metabolism | 124965.31 | 136316.06 | 97164.17 |
| Metabolism | Global and overview maps | ko01220 | Degradation of aromatic compounds | 122748.66 | 177641.37 | 56205.64 |
| Metabolism | Amino acid metabolism | ko00350 | Tyrosine metabolism | 143548.64 | 150044.61 | 61610.57 |
| Cellular Processes | Cellular community - prokaryotes | ko02025 | Biofilm formation - Pseudomonas aeruginosa | 139766.66 | 163614.83 | 48447.49 |
| Pathway level1 | Pathway level2 | Pathway level3 | Description | FG | MG | HG |
|  |  |  |  | Relative abundance | | |
| Metabolism | Lipid metabolism | ko00071 | Fatty acid degradation | 130867.51 | 144401.26 | 74948.66 |
| Genetic Information Processing | Folding, sorting and degradation | ko04122 | Sulfur relay system | 126232.85 | 136142.5 | 83020.95 |
| Metabolism | Amino acid metabolism | ko00340 | Histidine metabolism | 111266.36 | 113252.67 | 114566.34 |
| Metabolism | Xenobiotics biodegradation and metabolism | ko00362 | Benzoate degradation | 105304.69 | 162335.97 | 51677.2 |
| Metabolism | Amino acid metabolism | ko00380 | Tryptophan metabolism | 112193.81 | 111096.13 | 62886.34 |
| Genetic Information Processing | Replication and repair | ko03420 | Nucleotide excision repair | 88657.2 | 95363.36 | 100509.94 |
| Metabolism | Energy metabolism | ko00195 | Photosynthesis | 95297.59 | 99027.79 | 85683.76 |
| Organismal Systems | Aging | ko04212 | Longevity regulating pathway - worm | 97800.48 | 103818.03 | 73450.53 |
| Metabolism | Biosynthesis of other secondary metabolites | ko00521 | Streptomycin biosynthesis | 87011.17 | 73996.09 | 109678.88 |
| Organismal Systems | Endocrine system | ko04922 | Glucagon signaling pathway | 93889.64 | 89508.72 | 84919.03 |
| Metabolism | Carbohydrate metabolism | ko00660 | C5-Branched dibasic acid metabolism | 88511.9 | 90566.31 | 81574.27 |
| Human Diseases | Cancer: overview | ko05230 | Central carbon metabolism in cancer | 93723.28 | 84409.67 | 80415.09 |
| Environmental Information Processing | Signal transduction | ko04066 | HIF-1 signaling pathway | 80333.41 | 80675.04 | 85682.38 |
| Human Diseases | Drug resistance: antimicrobial | ko01502 | Vancomycin resistance | 76942.07 | 78752.45 | 82068 |
| Metabolism | Biosynthesis of other secondary metabolites | ko00261 | Monobactam biosynthesis | 78260.26 | 86230.51 | 64667.69 |
| Metabolism | Carbohydrate metabolism | ko00053 | Ascorbate and aldarate metabolism | 93886.82 | 99950.58 | 30624.7 |
| Organismal Systems | Environmental adaptation | ko04626 | Plant-pathogen interaction | 76818.68 | 77256.55 | 60537.68 |
| Cellular Processes | Transport and catabolism | ko04146 | Peroxisome | 79267.67 | 71399.11 | 59334.4 |
| Metabolism | Metabolism of cofactors and vitamins | ko00740 | Riboflavin metabolism | 71977.31 | 78923.39 | 57692.36 |
| Human Diseases | Cardiovascular disease | ko05418 | Fluid shear stress and atherosclerosis | 71908.79 | 74559.64 | 61665.34 |
| Pathway level1 | Pathway level2 | Pathway level3 | Description | FG | MG | HG |
|  |  |  |  | Relative abundance | | |
| Human Diseases | Drug resistance: antineoplastic | ko01523 | Antifolate resistance | 60228.25 | 60358.95 | 62701.51 |
| Metabolism | Metabolism of other amino acids | ko00410 | beta-Alanine metabolism | 81711.72 | 88357.38 | 36250.24 |
| Human Diseases | Infectious disease: bacterial | ko05132 | Salmonella infection | 62970.78 | 54458.39 | 65071.94 |
| Metabolism | Metabolism of cofactors and vitamins | ko00750 | Vitamin B6 metabolism | 59057.96 | 65741.58 | 50136.17 |
| Metabolism | Biosynthesis of other secondary metabolites | ko00333 | Prodigiosin biosynthesis | 57738.18 | 62580.15 | 54494.27 |
| Human Diseases | Infectious disease: bacterial | ko05133 | Pertussis | 65452.58 | 84694.02 | 22466.64 |
| Metabolism | Metabolism of other amino acids | ko00460 | Cyanoamino acid metabolism | 60143.51 | 52785.88 | 58230.82 |
| Metabolism | Xenobiotics biodegradation and metabolism | ko00627 | Aminobenzoate degradation | 55448.94 | 77900.71 | 29510.48 |
| Genetic Information Processing | Transcription | ko03020 | RNA polymerase | 52558.51 | 54180.08 | 55326.86 |
| Organismal Systems | Endocrine system | ko03320 | PPAR signaling pathway | 50083.6 | 57645.89 | 46007.78 |
| Human Diseases | Infectious disease: bacterial | ko05134 | Legionellosis | 56101.5 | 48888.34 | 48220.8 |
| Metabolism | Metabolism of other amino acids | ko00430 | Taurine and hypotaurine metabolism | 51550.38 | 50305.56 | 50891.25 |
| Metabolism | Metabolism of terpenoids and polyketides | ko00523 | Polyketide sugar unit biosynthesis | 47747.76 | 40950.68 | 61451.01 |
| Human Diseases | Infectious disease: bacterial | ko05152 | Tuberculosis | 48813.12 | 46802.85 | 48447.79 |
| Cellular Processes | Cell growth and death | ko04217 | Necroptosis | 45710.45 | 48186.45 | 45393.24 |
| Metabolism | Metabolism of other amino acids | ko00471 | D-Glutamine and D-glutamate metabolism | 44881.62 | 48978.13 | 43875.76 |
| Organismal Systems | Immune system | ko04621 | NOD-like receptor signaling pathway | 48225.87 | 39744.53 | 49227.94 |
| Metabolism | Glycan biosynthesis and metabolism | ko00511 | Other glycan degradation | 38252.88 | 31427.42 | 61045.05 |
| Metabolism | Metabolism of other amino acids | ko00473 | D-Alanine metabolism | 43235.43 | 43317.56 | 42137.1 |
| Human Diseases | Cancer: overview | ko05200 | Pathways in cancer | 48099.09 | 55618.25 | 23755.9 |
|  |  |  |  |  |  |  |
| Pathway level1 | Pathway level2 | Pathway level3 | Description | FG | MG | HG |
|  |  |  |  | Relative abundance | | |
| Metabolism | Xenobiotics biodegradation and metabolism | ko00625 | Chloroalkane and chloroalkene degradation | 49172.38 | 45229.15 | 30344.57 |
| Organismal Systems | Aging | ko04213 | Longevity regulating pathway - multiple species | 47131.14 | 43803.36 | 35321.88 |
| Metabolism | Xenobiotics biodegradation and metabolism | ko00982 | Drug metabolism - cytochrome P450 | 46380.21 | 53018.16 | 23976.07 |
| Metabolism | Xenobiotics biodegradation and metabolism | ko00980 | Metabolism of xenobiotics by cytochrome P450 | 45777.32 | 53000.49 | 23967.73 |
| Metabolism | Biosynthesis of other secondary metabolites | ko00960 | Tropane, piperidine and pyridine alkaloid biosynthesis | 40632.37 | 41575.79 | 26998.23 |
| Metabolism | Metabolism of other amino acids | ko00440 | Phosphonate and phosphinate metabolism | 44106.19 | 48655.95 | 16244.74 |
| Metabolism | Biosynthesis of other secondary metabolites | ko00401 | Novobiocin biosynthesis | 37063.62 | 37231.98 | 34391.55 |
| Metabolism | Carbohydrate metabolism | ko00562 | Inositol phosphate metabolism | 37161.01 | 43115.34 | 25086.63 |
| Human Diseases | Drug resistance: antineoplastic | ko01524 | Platinum drug resistance | 38578.57 | 46630.5 | 19428.73 |
| Human Diseases | Infectious disease: bacterial | ko05131 | Shigellosis | 38881.75 | 44409.66 | 13311.15 |
| Human Diseases | Neurodegenerative disease | ko05016 | Huntington disease | 35853.59 | 35657.73 | 24454.7 |
| Metabolism | Metabolism of terpenoids and polyketides | ko00281 | Geraniol degradation | 35336.06 | 47716.11 | 9119.17 |
| Metabolism | Lipid metabolism | ko00072 | Synthesis and degradation of ketone bodies | 33489.9 | 39107.2 | 19021.9 |
| Environmental Information Processing | Signal transduction | ko04070 | Phosphatidylinositol signaling system | 29679.13 | 34504.59 | 26188.95 |
| Metabolism | Xenobiotics biodegradation and metabolism | ko00626 | Naphthalene degradation | 33158.61 | 36978.04 | 19900.61 |
| Pathway level1 | Pathway level2 | Pathway level3 | Description | FG | MG | HG |
|  |  |  |  | Relative abundance | | |
| Human Diseases | Cancer: overview | ko05204 | Chemical carcinogenesis | 32292.76 | 42412.4 | 10659.91 |
| Organismal Systems | Nervous system | ko04727 | GABAergic synapse | 30780.88 | 26639.41 | 30109.58 |
| Metabolism | Biosynthesis of other secondary metabolites | ko00525 | Acarbose and validamycin biosynthesis | 28331.91 | 26104.44 | 30901.45 |
| Environmental Information Processing | Signal transduction | ko04068 | FoxO signaling pathway | 32904.88 | 30420.4 | 21725.15 |
| Human Diseases | Cancer: specific types | ko05225 | Hepatocellular carcinoma | 31247.23 | 41866.71 | 9528.71 |
| Human Diseases | Endocrine and metabolic disease | ko04931 | Insulin resistance | 27252.59 | 28279.76 | 26334.64 |
| Environmental Information Processing | Signal transduction | ko04016 | MAPK signaling pathway - plant | 29601.26 | 28585.52 | 23461.09 |
| Organismal Systems | Aging | ko04211 | Longevity regulating pathway | 32178.64 | 28454.1 | 20643.72 |
| Genetic Information Processing | Translation | ko03013 | RNA transport | 27200.64 | 26454.36 | 26904 |
| Metabolism | Xenobiotics biodegradation and metabolism | ko00622 | Xylene degradation | 25040.73 | 37104.01 | 13012.88 |
| Organismal Systems | Nervous system | ko04724 | Glutamatergic synapse | 25883.5 | 21034.91 | 26365.02 |
| Cellular Processes | Cell growth and death | ko04216 | Ferroptosis | 23952.59 | 21661.68 | 26543.86 |
| Environmental Information Processing | Signal transduction | ko04152 | AMPK signaling pathway | 21946.48 | 24454.23 | 25184.17 |
| Metabolism | Metabolism of cofactors and vitamins | ko00785 | Lipoic acid metabolism | 23509.38 | 27373.06 | 20222.54 |
| Metabolism | Xenobiotics biodegradation and metabolism | ko00361 | Chlorocyclohexane and chlorobenzene degradation | 21885.45 | 38233.74 | 9517.78 |
| Metabolism | Biosynthesis of other secondary metabolites | ko00940 | Phenylpropanoid biosynthesis | 23523.96 | 19269.36 | 26838.65 |
| Human Diseases | Immune disease | ko05340 | Primary immunodeficiency | 25727.44 | 27077.57 | 14820.64 |
| Pathway level1 | Pathway level2 | Pathway level3 | Description | FG | MG | HG |
|  |  |  |  | Relative abundance | | |
| Human Diseases | Infectious disease: bacterial | ko05120 | Epithelial cell signaling in Helicobacter pylori infection | 22512.77 | 24798.56 | 18736.39 |
| Metabolism | Metabolism of terpenoids and polyketides | ko01051 | Biosynthesis of ansamycins | 22910.29 | 25618.15 | 18495.01 |
| Metabolism | Lipid metabolism | ko00600 | Sphingolipid metabolism | 15728.9 | 13455.07 | 36674.06 |
| Metabolism | Biosynthesis of other secondary metabolites | ko00966 | Glucosinolate biosynthesis | 20084.06 | 20748.42 | 24669.3 |
| Cellular Processes | Transport and catabolism | ko04142 | Lysosome | 20183.96 | 12971.39 | 32257.92 |
| Metabolism | Biosynthesis of other secondary metabolites | ko00998 | Biosynthesis of various secondary metabolites - part 2 | 22329.6 | 21757.32 | 20817.26 |
| Metabolism | Xenobiotics biodegradation and metabolism | ko00930 | Caprolactam degradation | 24199.51 | 34276.88 | 5005.47 |
| Human Diseases | Infectious disease: bacterial | ko05100 | Bacterial invasion of epithelial cells | 32680.43 | 29883.46 | 398.52 |
| Human Diseases | Infectious disease: bacterial | ko05150 | Staphylococcus aureus infection | 21595.96 | 28632.11 | 12543.15 |
| Human Diseases | Infectious disease: bacterial | ko05135 | Yersinia infection | 32446.08 | 29821.6 | 396.56 |
| Human Diseases | Neurodegenerative disease | ko05014 | Amyotrophic lateral sclerosis (ALS) | 23071.13 | 22446.86 | 16619.77 |
| Organismal Systems | Environmental adaptation | ko04714 | Thermogenesis | 20588.38 | 15878.63 | 25335.17 |
| Metabolism | Metabolism of terpenoids and polyketides | ko00903 | Limonene and pinene degradation | 27099.17 | 26067.31 | 8121.09 |
| Organismal Systems | Endocrine system | ko04910 | Insulin signaling pathway | 20102.19 | 23638.92 | 16957.33 |
| Metabolism | Xenobiotics biodegradation and metabolism | ko00643 | Styrene degradation | 21198.44 | 29345.57 | 9433.34 |
| Metabolism | Biosynthesis of other secondary metabolites | ko00332 | Carbapenem biosynthesis | 18173.89 | 22869.54 | 18117.88 |
| Pathway level1 | Pathway level2 | Pathway level3 | Description | FG | MG | HG |
|  |  |  |  | Relative abundance | | |
| Metabolism | Xenobiotics biodegradation and metabolism | ko00623 | Toluene degradation | 15571.33 | 36598.34 | 6306.98 |
| Metabolism | Xenobiotics biodegradation and metabolism | ko00633 | Nitrotoluene degradation | 20611.77 | 22254.01 | 15878.53 |
| Metabolism | Biosynthesis of other secondary metabolites | ko00950 | Isoquinoline alkaloid biosynthesis | 19911.96 | 23396.69 | 15024.49 |
| Human Diseases | Endocrine and metabolic disease | ko04940 | Type I diabetes mellitus | 19091.26 | 18451.11 | 19185.17 |
| Cellular Processes | Cellular community - eukaryotes | ko04510 | Focal adhesion | 0.67 | 0.033 | 0.033 |
| Cellular Processes | Transport and catabolism | ko04145 | Phagosome | 0.67 | 0.033 | 0.033 |
| Environmental Information Processing | Signal transduction | ko04013 | MAPK signaling pathway - fly | 21106.75 | 20344.61 | 13085.05 |
| Metabolism | Glycan biosynthesis and metabolism | ko00531 | Glycosaminoglycan degradation | 16187.59 | 16326.48 | 22002.49 |
| Metabolism | Metabolism of cofactors and vitamins | ko00830 | Retinol metabolism | 20496.26 | 19372.5 | 14612.31 |
| Human Diseases | Cancer: overview | ko05206 | MicroRNAs in cancer | 20603.72 | 20483.41 | 13298.76 |
| Metabolism | Lipid metabolism | ko00121 | Secondary bile acid biosynthesis | 17070.45 | 22217.59 | 15085.86 |
| Metabolism | Lipid metabolism | ko01040 | Biosynthesis of unsaturated fatty acids | 20514.04 | 26709.93 | 6781.64 |
| Organismal Systems | Endocrine system | ko04918 | Thyroid hormone synthesis | 19406.64 | 21231.85 | 13332.56 |
| Organismal Systems | Endocrine system | ko04920 | Adipocytokine signaling pathway | 16529.79 | 14815.5 | 22267.78 |
| Human Diseases | Neurodegenerative disease | ko05010 | Alzheimer disease | 17320.46 | 17080.05 | 18145.16 |
| Human Diseases | Endocrine and metabolic disease | ko04930 | Type II diabetes mellitus | 18070.59 | 15686.11 | 17054.15 |
| Human Diseases | Cancer: overview | ko05203 | Viral carcinogenesis | 18086.01 | 15651.27 | 16174.73 |
| Human Diseases | Infectious disease: viral | ko05165 | Human papillomavirus infection | 18056.84 | 15635.6 | 16174.73 |
| Metabolism | Biosynthesis of other secondary metabolites | ko00524 | Neomycin, kanamycin and gentamicin biosynthesis | 16266.73 | 12139.24 | 21244.06 |
| Pathway level1 | Pathway level2 | Pathway level3 | Description | FG | MG | HG |
|  |  |  |  | Relative abundance | | |
| Human Diseases | Infectious disease: bacterial | ko05130 | Pathogenic Escherichia coli infection | 14615.78 | 16237.69 | 15837.42 |
| Genetic Information Processing | Folding, sorting and degradation | ko04141 | Protein processing in endoplasmic reticulum | 12745.5 | 8965.47 | 24790.83 |
| Metabolism | Glycan biosynthesis and metabolism | ko00603 | Glycosphingolipid biosynthesis - globo and isoglobo series | 11515.54 | 10267.43 | 23541.56 |
| Metabolism | Biosynthesis of other secondary metabolites | ko00405 | Phenazine biosynthesis | 15048.38 | 14614.44 | 14312.82 |
| Metabolism | Lipid metabolism | ko00592 | alpha-Linolenic acid metabolism | 17624.44 | 18652.97 | 6724.37 |
| Metabolism | Metabolism of terpenoids and polyketides | ko01055 | Biosynthesis of vancomycin group antibiotics | 14261.33 | 12891.12 | 15807.18 |
| Metabolism | Xenobiotics biodegradation and metabolism | ko00364 | Fluorobenzoate degradation | 10527.44 | 28163.57 | 2527.93 |
| Metabolism | Metabolism of terpenoids and polyketides | ko01053 | Biosynthesis of siderophore group nonribosomal peptides | 14980.67 | 16573.27 | 9373.61 |
| Cellular Processes | Cell growth and death | ko04214 | Apoptosis - fly | 12919.64 | 15447.22 | 11815.59 |
| Metabolism | Lipid metabolism | ko00590 | Arachidonic acid metabolism | 12727.01 | 17132.55 | 10143.91 |
| Metabolism | Xenobiotics biodegradation and metabolism | ko00621 | Dioxin degradation | 14880.64 | 14663.9 | 7030.46 |
| Metabolism | Metabolism of terpenoids and polyketides | ko00908 | Zeatin biosynthesis | 10598.33 | 12086.01 | 13107.69 |
| Metabolism | Glycan biosynthesis and metabolism | ko00513 | Various types of N-glycan biosynthesis | 9780.08 | 9268.62 | 15499.45 |
| Human Diseases | Cancer: overview | ko05205 | Proteoglycans in cancer | 10084.24 | 12007.84 | 11453.35 |
| Metabolism | Glycan biosynthesis and metabolism | ko00604 | Glycosphingolipid biosynthesis - ganglio series | 9747.08 | 9232.97 | 14519.64 |
| Pathway level1 | Pathway level2 | Pathway level3 | Description | FG | MG | HG |
|  |  |  |  | Relative abundance | | |
| Metabolism | Xenobiotics biodegradation and metabolism | ko00791 | Atrazine degradation | 12199.49 | 18824.93 | 2215.09 |
| Metabolism | Metabolism of terpenoids and polyketides | ko01054 | Nonribosomal peptide structures | 13585.48 | 13330.44 | 5042.68 |
| Organismal Systems | Excretory system | ko04964 | Proximal tubule bicarbonate reclamation | 11459.87 | 12198.18 | 6948.25 |
| Environmental Information Processing | Signal transduction | ko04151 | PI3K-Akt signaling pathway | 8210.52 | 9518.92 | 10190.94 |
| Metabolism | Xenobiotics biodegradation and metabolism | ko00642 | Ethylbenzene degradation | 12316.37 | 12071.33 | 2915.74 |
| Environmental Information Processing | Signal transduction | ko04011 | MAPK signaling pathway - yeast | 11071.89 | 8109.49 | 7558.67 |
| Human Diseases | Infectious disease: parasitic | ko05143 | African trypanosomiasis | 10903.43 | 12863.73 | 1552.08 |
| Organismal Systems | Immune system | ko04657 | IL-17 signaling pathway | 7482.77 | 7552.62 | 9109.51 |
| Organismal Systems | Immune system | ko04612 | Antigen processing and presentation | 7481.94 | 7552.62 | 9109.51 |
| Organismal Systems | Endocrine system | ko04915 | Estrogen signaling pathway | 7481.94 | 7552.62 | 9109.51 |
| Organismal Systems | Endocrine system | ko04914 | Progesterone-mediated oocyte maturation | 7481.94 | 7552.62 | 9109.51 |
| Human Diseases | Cancer: specific types | ko05215 | Prostate cancer | 7481.94 | 7552.62 | 9109.51 |
| Organismal Systems | Immune system | ko04659 | Th17 cell differentiation | 7481.94 | 7552.62 | 9109.51 |
| Metabolism | Lipid metabolism | ko00565 | Ether lipid metabolism | 9023.37 | 11421.42 | 3591.65 |
| Organismal Systems | Digestive system | ko04973 | Carbohydrate digestion and absorption | 9043.66 | 10066.91 | 4506.02 |
| Metabolism | Glycan biosynthesis and metabolism | ko00572 | Arabinogalactan biosynthesis - Mycobacterium | 9896.46 | 9282.67 | 4398.69 |
| Human Diseases | Infectious disease: parasitic | ko05146 | Amoebiasis | 9195.07 | 9776.3 | 3809.78 |
| Human Diseases | Endocrine and metabolic disease | ko04932 | Non-alcoholic fatty liver disease (NAFLD) | 4935.05 | 1638.21 | 3921.03 |
| Pathway level1 | Pathway level2 | Pathway level3 | Description | FG | MG | HG |
|  |  |  |  | Relative abundance | | |
| Organismal Systems | Digestive system | ko04972 | Pancreatic secretion | 9029.08 | 10016.4 | 3626.6 |
| Organismal Systems | Endocrine system | ko04917 | Prolactin signaling pathway | 8678.18 | 9792.83 | 4045.63 |
| Metabolism | Lipid metabolism | ko00120 | Primary bile acid biosynthesis | 7320.6 | 8436.9 | 6185.89 |
| Human Diseases | Infectious disease: parasitic | ko05142 | Chagas disease (American trypanosomiasis) | 10626.92 | 9056.11 | 1444.09 |
| Human Diseases | Cancer: overview | ko05231 | Choline metabolism in cancer | 6798.73 | 7540.49 | 6550.89 |
| Organismal Systems | Development and regeneration | ko04361 | Axon regeneration | 6808.13 | 7479.51 | 6180.8 |
| Environmental Information Processing | Signal transduction | ko04072 | Phospholipase D signaling pathway | 6714.91 | 7479.18 | 6180.8 |
| Metabolism | Biosynthesis of other secondary metabolites | ko00965 | Betalain biosynthesis | 7320.15 | 11088.55 | 1848.73 |
| Metabolism | Lipid metabolism | ko00591 | Linoleic acid metabolism | 7036.69 | 9049.63 | 3580.33 |
| Human Diseases | Endocrine and metabolic disease | ko04934 | Cushing syndrome | 7325.25 | 7423.45 | 3505.2 |
| Human Diseases | Cancer: specific types | ko05211 | Renal cell carcinoma | 7325.25 | 7423.45 | 3505.2 |
| Metabolism | Lipid metabolism | ko00140 | Steroid hormone biosynthesis | 5189.99 | 7686.54 | 4808.11 |
| Metabolism | Metabolism of terpenoids and polyketides | ko00981 | Insect hormone biosynthesis | 8192.07 | 4623.87 | 4072.73 |
| Organismal Systems | Digestive system | ko04974 | Protein digestion and absorption | 4014.53 | 1749.19 | 5975.22 |
| Metabolism | Xenobiotics biodegradation and metabolism | ko00624 | Polycyclic aromatic hydrocarbon degradation | 3200.43 | 6749.54 | 1785.85 |
| Metabolism | Glycan biosynthesis and metabolism | ko00510 | N-Glycan biosynthesis | 2877.21 | 1327.27 | 7321.3 |
| Human Diseases | Cancer: specific types | ko05219 | Bladder cancer | 5170.74 | 4781.94 | 1078.9 |
| Metabolism | Biosynthesis of other secondary metabolites | ko00311 | Penicillin and cephalosporin biosynthesis | 4163.91 | 3150.9 | 3372.01 |
|  |  |  |  |  |  |  |
| Pathway level1 | Pathway level2 | Pathway level3 | Description | FG | MG | HG |
|  |  |  |  | Relative abundance | | |
| Human Diseases | Neurodegenerative disease | ko05012 | Parkinson disease | 4934.78 | 1638.21 | 3921.03 |
| Cellular Processes | Cell growth and death | ko04210 | Apoptosis | 3193.46 | 2734.22 | 4301.24 |
| Organismal Systems | Endocrine system | ko04614 | Renin-angiotensin system | 3467.89 | 3127.91 | 2728.89 |
| Genetic Information Processing | Replication and repair | ko03450 | Non-homologous end-joining | 4858.44 | 1613.84 | 1352.06 |
| Organismal Systems | Digestive system | ko04978 | Mineral absorption | 1969.22 | 2389.51 | 2471.15 |
| Organismal Systems | Endocrine system | ko04919 | Thyroid hormone signaling pathway | 2123.86 | 4381.1 | 25.5 |
| Human Diseases | Neurodegenerative disease | ko05020 | Prion diseases | 2309.44 | 3113.58 | 770.09 |
| Organismal Systems | Circulatory system | ko04260 | Cardiac muscle contraction | 2750.93 | 971.08 | 2290.72 |
| Metabolism | Xenobiotics biodegradation and metabolism | ko00984 | Steroid degradation | 4890.5 | 811.48 | 10.58 |
| Metabolism | Metabolism of terpenoids and polyketides | ko00906 | Carotenoid biosynthesis | 3469.17 | 1120.36 | 875.26 |
| Cellular Processes | Cell growth and death | ko04113 | Meiosis - yeast | 2657.28 | 251.74 | 2485.59 |
| Human Diseases | Infectious disease: viral | ko05164 | Influenza A | 2200.92 | 663.26 | 1612.48 |
| Human Diseases | Infectious disease: viral | ko05163 | Human cytomegalovirus infection | 2188.68 | 662.46 | 1612.48 |
| Human Diseases | Infectious disease: viral | ko05167 | Kaposi sarcoma-associated herpesvirus infection | 2188.01 | 662.46 | 1612.48 |
| Human Diseases | Cancer: specific types | ko05222 | Small cell lung cancer | 2188.01 | 662.46 | 1612.48 |
| Human Diseases | Infectious disease: viral | ko05168 | Herpes simplex virus 1 infection | 2187.85 | 662.46 | 1612.48 |
| Cellular Processes | Cell growth and death | ko04215 | Apoptosis - multiple species | 2187.18 | 662.46 | 1612.48 |
| Human Diseases | Cancer: specific types | ko05210 | Colorectal cancer | 2187.18 | 662.46 | 1612.48 |
| Human Diseases | Infectious disease: viral | ko05169 | Epstein-Barr virus infection | 2187.18 | 662.46 | 1612.48 |
| Human Diseases | Infectious disease: viral | ko05161 | Hepatitis B | 2187.18 | 662.46 | 1612.48 |
| Human Diseases | Infectious disease: viral | ko05160 | Hepatitis C | 2187.18 | 662.46 | 1612.48 |
| Pathway level1 | Pathway level2 | Pathway level3 | Description | FG | MG | HG |
|  |  |  |  | Relative abundance | | |
| Human Diseases | Infectious disease: viral | ko05170 | Human immunodeficiency virus 1 infection | 2187.18 | 662.46 | 1612.48 |
| Human Diseases | Infectious disease: viral | ko05162 | Measles | 2187.18 | 662.46 | 1612.48 |
| Human Diseases | Infectious disease: parasitic | ko05145 | Toxoplasmosis | 2187.18 | 662.46 | 1612.48 |
| Human Diseases | Cardiovascular disease | ko05416 | Viral myocarditis | 2187.18 | 662.46 | 1612.48 |
| Cellular Processes | Cell growth and death | ko04115 | p53 signaling pathway | 2187.18 | 662.46 | 1612.48 |
| Cellular Processes | Transport and catabolism | ko04138 | Autophagy - yeast | 1664.21 | 565.22 | 1080.41 |
| Metabolism | Glycan biosynthesis and metabolism | ko00571 | Lipoarabinomannan (LAM) biosynthesis | 2532.97 | 174.17 | 347.63 |
| Human Diseases | Infectious disease: bacterial | ko05110 | Vibrio cholerae infection | 1315.33 | 1191 | 180.67 |
| Environmental Information Processing | Signal transduction | ko04071 | Sphingolipid signaling pathway | 389.08 | 1901.13 | 0.033 |
| Human Diseases | Immune disease | ko05322 | Systemic lupus erythematosus | 330.95 | 1905.08 | 0.75 |
| Organismal Systems | Immune system | ko04622 | RIG-I-like receptor signaling pathway | 847.15 | 633.68 | 680.39 |
| Organismal Systems | Endocrine system | ko04916 | Melanogenesis | 241.66 | 1908.41 | 7.42 |
| Metabolism | Metabolism of other amino acids | ko00472 | D-Arginine and D-ornithine metabolism | 880.43 | 105.2 | 480.6 |
| Genetic Information Processing | Translation | ko03008 | Ribosome biogenesis in eukaryotes | 16113.46 | 19074.25 | 13666.7 |
| Metabolism | Metabolism of terpenoids and polyketides | ko00253 | Tetracycline biosynthesis | 872.19 | 526.5 | 0.033 |
| Genetic Information Processing | Folding, sorting and degradation | ko03050 | Proteasome | 635 | 31.76 | 339.91 |
| Genetic Information Processing | Transcription | ko03022 | Basal transcription factors | 433.2 | 30.74 | 363.91 |
| Human Diseases | Substance dependence | ko05034 | Alcoholism | 770.03 | 37.34 | 8.34 |
| Organismal Systems | Nervous system | ko04728 | Dopaminergic synapse | 747.03 | 21.67 | 8.34 |
| Organismal Systems | Nervous system | ko04726 | Serotonergic synapse | 740.19 | 21.67 | 8.34 |
| Human Diseases | Substance dependence | ko05031 | Amphetamine addiction | 739.36 | 21.67 | 8.34 |
| Human Diseases | Substance dependence | ko05030 | Cocaine addiction | 739.36 | 21.67 | 8.34 |
| Pathway level1 | Pathway level2 | Pathway level3 | Description | FG | MG | HG |
|  |  |  |  | Relative abundance | | |
| Environmental Information Processing | Signal transduction | ko04024 | cAMP signaling pathway | 401.66 | 22.09 | 343.25 |
| Metabolism | Biosynthesis of other secondary metabolites | ko00941 | Flavonoid biosynthesis | 3.67 | 48.33 | 574.99 |
| Metabolism | Biosynthesis of other secondary metabolites | ko00945 | Stilbenoid, diarylheptanoid and gingerol biosynthesis | 3.67 | 48.33 | 574.99 |
| Human Diseases | Neurodegenerative disease | ko05017 | Spinocerebellar ataxia | 0.033 | 4.66 | 350.66 |
| Organismal Systems | Endocrine system | ko04928 | Parathyroid hormone synthesis, secretion and action | 230 | 111.67 | 7.67 |
| Metabolism | Biosynthesis of other secondary metabolites | ko00943 | Isoflavonoid biosynthesis | 191.5 | 108 | 7.67 |
| Metabolism | Biosynthesis of other secondary metabolites | ko00404 | Staurosporine biosynthesis | 243.82 | 17.17 | 40.02 |
| Metabolism | Xenobiotics biodegradation and metabolism | ko00363 | Bisphenol degradation | 251.44 | 4 | 0.033 |
| Metabolism | Lipid metabolism | ko00100 | Steroid biosynthesis | 228.34 | 13.01 | 13.33 |
| Human Diseases | Cardiovascular disease | ko05410 | Hypertrophic cardiomyopathy (HCM) | 215.17 | 21.34 | 6.67 |
| Metabolism | Biosynthesis of other secondary metabolites | ko00232 | Caffeine metabolism | 214.93 | 13.34 | 1.67 |
| Organismal Systems | Endocrine system | ko04924 | Renin secretion | 183.5 | 8.67 | 6.67 |
| Organismal Systems | Immune system | ko04640 | Hematopoietic cell lineage | 169.33 | 6.67 | 0.033 |
| Metabolism | Biosynthesis of other secondary metabolites | ko00901 | Indole alkaloid biosynthesis | 133.55 | 4 | 0.033 |
|  |  |  |  |  |  |  |
| Pathway level1 | Pathway level2 | Pathway level3 | Description | FG | MG | HG |
|  |  |  |  | Relative abundance | | |
| Metabolism | Metabolism of terpenoids and polyketides | ko00909 | Sesquiterpenoid and triterpenoid biosynthesis | 3.68 | 10.67 | 62.66 |
| Metabolism | Xenobiotics biodegradation and metabolism | ko00365 | Furfural degradation | 72.64 | 0.033 | 0.033 |
| Cellular Processes | Transport and catabolism | ko04144 | Endocytosis | 57.54 | 8.33 | 0.96 |
| Metabolism | Biosynthesis of other secondary metabolites | ko00997 | Biosynthesis of various secondary metabolites - part 3 | 61.23 | 0.8 | 0.033 |
| Organismal Systems | Immune system | ko04666 | Fc gamma R-mediated phagocytosis | 52.73 | 4.47 | 0.033 |
| Metabolism | Lipid metabolism | ko00062 | Fatty acid elongation | 50.01 | 0.033 | 0.033 |
| Metabolism | Metabolism of terpenoids and polyketides | ko01057 | Biosynthesis of type II polyketide products | 38.71 | 7.83 | 0.033 |
| Human Diseases | Cardiovascular disease | ko05414 | Dilated cardiomyopathy (DCM) | 31.67 | 12.67 | 0.033 |
| Organismal Systems | Endocrine system | ko04912 | GnRH signaling pathway | 40.33 | 3.67 | 0.033 |
| Human Diseases | Cancer: specific types | ko05212 | Pancreatic cancer | 40.33 | 3.67 | 0.033 |
| Environmental Information Processing | Signal transduction | ko04014 | Ras signaling pathway | 40.33 | 3.67 | 0.033 |
| Organismal Systems | Digestive system | ko04976 | Bile secretion | 30.34 | 9.08 | 0.58 |
| Human Diseases | Cancer: specific types | ko05226 | Gastric cancer | 36.33 | 0.033 | 0.033 |
| Metabolism | Biosynthesis of other secondary metabolites | ko00944 | Flavone and flavonol biosynthesis | 25.44 | 3.06 | 0.67 |
| Organismal Systems | Nervous system | ko04723 | Retrograde endocannabinoid signaling | 0.83 | 4.67 | 17.83 |
| Organismal Systems | Excretory system | ko04961 | Endocrine and other factor-regulated calcium reabsorption | 75 | 3.67 | 0.33 |
| Organismal Systems | Endocrine system | ko04921 | Oxytocin signaling pathway | 0.83 | 0.033 | 0.033 |
| Pathway level1 | Pathway level2 | Pathway level3 | Description | FG | MG | HG |
|  |  |  |  | Relative abundance | | |
| Cellular Processes | Cellular community - eukaryotes | ko04520 | Adherens junction | 12.4 | 0.8 | 0.033 |
| Metabolism | Metabolism of terpenoids and polyketides | ko01052 | Type I polyketide structures | 11.5 | 0.033 | 6.67 |
| Organismal Systems | Immune system | ko04062 | Chemokine signaling pathway | 12.4 | 0.8 | 0.033 |
| Cellular Processes | Cellular community - eukaryotes | ko04530 | Tight junction | 12.4 | 0.8 | 0.033 |
| Organismal Systems | Development and regeneration | ko04380 | Osteoclast differentiation | 12 | 0.033 | 0.033 |
| Human Diseases | Immune disease | ko05323 | Rheumatoid arthritis | 11.33 | 0.033 | 0.033 |
| Environmental Information Processing | Signal transduction | ko04020 | Calcium signaling pathway | 3.33 | 0.033 | 6.67 |
| Metabolism | Metabolism of terpenoids and polyketides | ko01059 | Biosynthesis of enediyne antibiotics | 7 | 0.033 | 0.033 |
| Metabolism | Metabolism of terpenoids and polyketides | ko00522 | Biosynthesis of 12-, 14- and 16-membered macrolides | 4.83 | 0.033 | 0.033 |
| Genetic Information Processing | Translation | ko03015 | mRNA surveillance pathway | 1.83 | 0.033 | 0.033 |
| Organismal Systems | Immune system | ko04624 | Toll and Imd signaling pathway | 1.33 | 0.033 | 0.033 |
| Organismal Systems | Immune system | ko04625 | C-type lectin receptor signaling pathway | 0.83 | 0.033 | 0.033 |
| Organismal Systems | Endocrine system | ko04911 | Insulin secretion | 0.83 | 0.033 | 0.033 |
| Human Diseases | Infectious disease: parasitic | ko05140 | Leishmaniasis | 0.83 | 0.033 | 0.033 |
| Environmental Information Processing | Signal transduction | ko04064 | NF-kappa B signaling pathway | 0.83 | 0.033 | 0.033 |
| Organismal Systems | Endocrine system | ko04923 | Regulation of lipolysis in adipocytes | 0.83 | 0.033 | 0.033 |
| Environmental Information Processing | Signal transduction | ko04668 | TNF signaling pathway | 0.83 | 0.033 | 0.033 |
|  |  |  |  |  |  |  |
|  |  |  |  |  |  |  |
| Pathway level1 | Pathway level2 | Pathway level3 | Description | FG | MG | HG |
|  |  |  |  | Relative abundance | | |
| Human Diseases | Cardiovascular disease | ko05412 | Arrhythmogenic right ventricular cardiomyopathy (ARVC) | 0.67 | 0.033 | 0.033 |
| Environmental Information Processing | Signal transduction | ko04370 | VEGF signaling pathway | 0.83 | 0.033 | 0.033 |
| Metabolism | Biosynthesis of other secondary metabolites | ko00999 | Biosynthesis of various secondary metabolites - part 1 | 0.67 | 0.033 | 0.033 |
| Environmental Information Processing | Signaling molecules and interaction | ko04512 | ECM-receptor interaction | 0.67 | 0.033 | 0.033 |
| Environmental Information Processing | Signaling molecules and interaction | ko04080 | Neuroactive ligand-receptor interaction | 0.67 | 0.033 | 0.033 |
| Environmental Information Processing | Signal transduction | ko04015 | Rap1 signaling pathway | 0.67 | 0.033 | 0.033 |

Relative abundance of predicted functions. Relative abundance refers to the abundance of functions in each group corresponding to level 3 of the KEGG pathway.

**Supplementary Table 5** Comparison of relative abundance of gut microbiota function at KEGG pathways (level 1, 2 and 3) with significant differences.

| Fraction | KEGG Level-1 and Level-2 | KEGG Level-3 | F | *P* |
| --- | --- | --- | --- | --- |
| Foregut | Environmental Information Processing; Signal transduction | MAPK signaling pathway - fly | 5.606 | 0.0424 |
|  | Metabolism; Glycan biosynthesis and metabolism | Arabinogalactan biosynthesis - Mycobacterium | 14.97 | 0.0047 |
| Midgut | Environmental information processing; Membrane transport | Phosphotransferase system (PTS) | 7.127 | 0.0260 |
|  | Metabolism; Carbohydrate metabolism | Propanoate metabolism | 8.438 | 0.0180 |
|  | Metabolism; Metabolism of terpenoids and polyketides | Geraniol degradation | 6.676 | 0.0298 |
|  | Human Diseases; Drug resistance:antimicrobial | beta-Lactam resistance | 6.210 | 0.0346 |
|  | Metabolism; Metabolism of other amino acids | Glutathione metabolism | 11.89 | 0.0082 |
|  | Metabolism; Amino acid metabolism | Phenylalanine metabolism | 6.119 | 0.0356 |
|  | Metabolism; Amino acid metabolism | Tyrosine metabolism | 8.437 | 0.0180 |
|  | Metabolism; Metabolism of other amino acids | beta-Alanine metabolism | 5.983 | 0.0373 |
|  | Human Diseases; Cancer: overview | Pathways in cancer | 5.848 | 0.0390 |
|  | Human Diseases; Cancer: overview | Chemical carcinogenesis | 5.936 | 0.0378 |
|  | Human Diseases; Cancer: specific types | Hepatocellular carcinoma | 5.726 | 0.0406 |
|  | Human Diseases; Immune disease | Primary immunodeficiency | 12.16 | 0.0078 |
|  | Metabolism; Lipid metabolism | Biosynthesis of unsaturated fatty acids | 7.622 | 0.0225 |
|  | Metabolism; Metabolism of terpenoids and polyketides | Nonribosomal peptide structures | 5.460 | 0.0446 |
|  | Metabolism; Xenobiotics biodegradation and metabolism | Ethylbenzene degradation | 5.513 | 0.0438 |
|  | Human Diseases; Infectious disease: parasitic | African trypanosomiasis | 6.901 | 0.0278 |
|  | Human Diseases; Endocrine and metabolic disease | Cushing syndrome | 8.115 | 0.0197 |
|  | Human Diseases; Cancer: specific types | Renal cell carcinoma | 8.115 | 0.0197 |
| Hindgut | Metabolism; Glycan biosynthesis and metabolism | Other glycan degradation | 13.26 | 0.0063 |
|  | Metabolism; Lipid metabolism | Sphingolipid metabolism | 11.11 | 0.0096 |
|  | Cellular Processes; Transport and catabolism | Lysosome | 19.10 | 0.0025 |
|  | Metabolism; Glycan biosynthesis and metabolism | N-Glycan biosynthesis | 11.84 | 0.0083 |
|  | KEGG Level-1 and Level-2 | KEGG Level-3 | F | *P* |
|  | Genetic Information Processing; Folding, sorting and degradation | Protein processing in endoplasmic reticulum | 8.179 | 0.0193 |
|  | Metabolism; Glycan biosynthesis and metabolism | Glycosphingolipid biosynthesis-globo and isoglobo series | 27.79 | 0.0009 |
|  | Metabolism; Glycan biosynthesis and metabolism | Various types of N-glycan biosynthesis | 11.84 | 0.0083 |
|  | Metabolism; Glycan biosynthesis and metabolism | Glycosphingolipid biosynthesis - ganglio series | 8.844 | 0.0163 |

Significant differences (p < 0.05) were determined by one-way ANOVA analysis of variance with 2 degrees of freedom (*df* = 2). F: F value is the level of significant difference, the greater the F value, the more significant. The *P* value is an indicator to measure the difference. The *P* < 0.05 indicating that there is a significant difference between the two groups. The *P* < 0.01 indicating that the difference between the two groups is extremely significant.

**Supplementary Table 6** Comparison of relative abundance of gut microbiota function at KEGG pathway level 2 with significant differences of gut flores.

| KEGG Level-2 | F | *P* |
| --- | --- | --- |
| Membrane transport | 6.747 | 0.0292 |
| Metabolism of other amino acids | 6.739 | 0.0292 |
| Cancer: overview | 5.364 | 0.0461 |
| Cancer: specific types | 7.039 | 0.0267 |
| Infectious disease: parasitic | 6.779 | 0.0289 |
| Immune disease | 9.123 | 0.0152 |
| Cellular community-eukaryotes | 5.371 | 0.0460 |

Significant differences (p < 0.05) were determined by one-way ANOVA analysis of variance with 2 degrees of freedom (*df* = 2). F: F value is the level of significant difference, the greater the F value, the more significant. The *P* value is an indicator to measure the difference. The *P* < 0.05 indicating that there is a significant difference between the two groups. The *P* < 0.01 indicating that the difference between the two groups is extremely significant.

**Supplementary Table 7** Blast-based alignment of 16S rRNA from the midgut of the final-instar larva of *P. xanthodes* with the GenBank database.

| Isolated strains | GenBank Accession No. | Closest related species  (Accession No.) | Identity/% |
| --- | --- | --- | --- |
| PX1-2 | *OP782094* | *Bacillus subtilis subsp.*（*MN704470.1*） | 99.51% |
| PX2-4 | *OP782095* | *Enterococcus faecalis*（*MN548681*） | 99.52% |
| PX3-5 | *OP782096* | *Hafnia alvei*(*CP066284.1*) | 99.72% |
| PX4-6 | *OP782097* | *Uncultured Bacillus sp.*( *MG825091.1*) | 99.45% |
| PX5-8 | *OP782098* | *Bacillus sp.*( *MG309326.1*) | 99.45% |
| PX6-10 | *OP782099* | *Lysinibacillus fusiformis*(*MF662437.1*) | 98.91% |
| PX7-17 | *OP7820100* | *Raoultella ornithinolytica*(*MT568560.1*) | 99.65% |
| PX8-37 | *OP7820101* | *Citrobacter sp.*( *LR699014.1*) | 99.93% |
| PX9-13 | *OP7820102* | *Oceanobacillus profundus*(*HQ595230.1*) | 99.45% |
| PX10-14 | *OP7820103* | *Aeromonas hydrophila*(*ON203004.1*) | 99.72% |
| PX11-15 | *OP7820104* | *Bacillus megaterium*(*KY495205.1*) | 99.58% |
| PX12-19 | *OP7820105* | *Morganella morganii*(*MG654672.1*) | 99.72% |
| PX13-44 | *OP7820106* | *Gordonia sp.*( *CP070351.1*) | 99.79% |
| PX14-23 | *OP7820107* | *Microbacterium oxydans*(*MT533951.1*) | 99.93% |
| PX15-25 | *OP7820108* | *Uncultured bacterium clone*(*MF092516.1*) | 99.65% |
| PX16-42 | *OP7820109* | *Lactobacillus sakei*(*MT463883.1*) | 99.86% |
| PX17-41 | *OP777412* | *Staphylococcus hominis subsp*(*MN428234.1*) | 98.69% |
| PX18-40 | *OP777413* | *Lactococcus garvieae*(*MT597707.1*) | 99.51% |
| PX19-31 | *OP777414* | *Fictibacillus sp.*( *KY385631.3*) | 99.17% |
| PX20-1 | *OP777415* | *Bacillus velezensis*(*MN493079.1*) | 98.09% |
| PX21-34 | *OP777416* | *Enterococcus thailandicus*(*LT223669.1*) | 99.79% |
| PX22-36 | *OP777417* | *Sphingobacterium sp.*( *KJ152099.1*) | 99.79% |
| PX23-2 | *OP777418* | *Acinetobacter sp.*（*MN577382.1*） | 99.65% |
| PX24-43 | *OP777419* | *Flavobacterium sp.*( *MF405112.1*) | 99.17% |
| PX25-33 | *OP777420* | *Rhodococcus sp.*( *MW007892.1*) | 99.36% |
| PX26-54 | *OP777421* | *Bacillus firmus*(*KF228909*) | 99.86% |

Isolated strains: Isolate strain number; GenBank Accession No: Genbank registry number obtained from isolated strains; Closest related species (Accession No): the species in NCBI GenBank.
